# Evaluating the physiological responses and identifying stress tolerance of Akabare chili landraces to individual and combined drought and heat stresses

**DOI:** 10.1093/aobpla/plad083

**Published:** 2023-11-25

**Authors:** Damodar Poudyal, Bal Krishna Joshi, Rong Zhou, Carl-Otto Ottosen, Kishor Chandra Dahal

**Affiliations:** Postgraduate Program, Institute of Agriculture and Animal Science, Tribhuvan University, Kirtipur-10, 44618 Kathmandu, Nepal; National Agriculture Genetic Resource Center, Nepal Agriculture Research Council, Khumaltar, 44700 Lalitpur, Nepal; College of Horticulture, Nanjing Agriculture University, Weigang No.1, 210095 Nanjing, China; Department of Food Science, Aarhus University, Agro Food Park 48, 8200 Aarhus N, Denmark; Postgraduate Program, Institute of Agriculture and Animal Science, Tribhuvan University, Kirtipur-10, 44618 Kathmandu, Nepal

**Keywords:** Biomass, leaf cooling, photosynthesis, stomata, stress tolerance

## Abstract

**Abstract**. Akabare chili (*Capsicum annuum*) contributes to Nepalese rural livelihoods but suffers from low productivity due to various abiotic stresses including drought and heat. This study aimed to assess the physiological responses of Akabare chili landraces to heat and drought stress, individually and together, and to identify stress-tolerant genotypes in the early vegetative stage. Selected eight Akabare chili landraces and chili variety ‘Jwala’ were subjected to control (30/22 °C day/night) and heat stress (40/32 °C) conditions with irrigation, and drought stress (30/22 °C) and combined drought-heat stress conditions without irrigation for 7 days, followed by a 5-day recovery under control condition. Stress-tolerant landraces showed better performance compared to sensitive ones in terms of efficacy of PS II (*F*_*v*_/*F*_*m*_), transpiration rate (*E*), net photosynthetic rate (*P*_N_), stomatal conductance (*g*_*s*_), leaf temperature depression, water use efficiency (WUE) and the ratio of stomata pore area to stomata area under stress conditions, resulting in improved biomass. Although all genotypes performed statistically similar under control conditions, their responses *F*_*v*_/*F*_*m*_, *P*_N_, *E*, *g*_*s*_ and WUE were significantly reduced under thermal stress, further reduced under drought stress, and severely declined under the combination of both. Total biomass exhibited a 57.48 % reduction due to combined stress, followed by drought (37.8 %) and heat (21.4 %) compared to the control. Among the landraces, C44 showed the most significant gain in biomass (35 %), followed by DKT77 (33.48 %), while the lowest gain percentage was observed for C64C and PPR77 during the recovery phase (29 %). The tolerant landraces also showed a higher percentage of leaf cooling, chlorophyll content and leaf relative water content with fewer stomata but broader openings of pores. The study identifies potential stress-tolerant Akabare chili landraces and discusses the stress-tolerant physiological mechanisms to develop resilient crop varieties in changing climates.

## Teaser

The Akabare chili landraces hold significant importance as a spice crop and support livelihoods in Nepal. This study marks the initial endeavor to phenotype Akabare chili landraces under conditions of drought, heat and their combination. The interplay of efficacy of PSII (*F_v_*/*F_m_*), net photosynthetic rate, intercellular CO_2_ concentration and transpiration rate is crucial for sustaining carbon gain. Across the studied genotypes, total biomass experienced a higher reduction (57.48 %) under combined stress, a milder reduction under drought (37.8 %) and a lower reduction under heat (21.4 %) stress compared to the control treatment. Akabare chili landraces C44, DKT77, and C45C demonstrated lesser compounded impacts of drought and heat on *F_v_*/*F_m_*, net photosynthetic rate, stomatal conductance, leaf relative water content and total biomass. These characteristics position them as promising candidates for future crop improvement programmes.

## Introduction

Chili (*Capsicum* spp.) has been a popular vegetable spice crop since the Columbian Exchange (Meghvansi *et al.* 2010). It is grown in tropical to warm temperate regions of the world as a cash crop. Out of five, three taxa of *Capsicum* species (*C. annuum, C. baccatum* and *C. frutescens*) have been reported to be commonly grown in Nepal during the spring to autumn season (Sugiyama *et al.* 2018; Poudyal *et al.* 2023*c*). Akabare chili (*Capsicum annuum* L.) is a widely cultivated chili landraces in the hilly regions of Nepal. It is typically grown between March and December, primarily relying on rain-fed conditions. Notably, the growth pattern of this crop ranges from being an annual to biennial and occasionally perennial, setting it apart from other common chili genotypes. Akabare chili is well known for its spiciness and fragrance (Malakar *et al.* 2019; Poudyal *et al.* 2023*b*). The eastern mid-hill region of Nepal is considered a major repository of Akabare chili due to the presence of wider genotypic variations in the area and the region occupies a major proportion (76 %) of the total hectarage. A recent field study has unveiled the socio-cultural importance of having Akabare chili in the Nepalese kitchen (Poudyal *et al.* 2023*c*) and the significant influence of Akabare chili landraces on households economy, indicating a substantial increase of 189 % in gross profits. This notable economic upswing can be attributed to the escalating demand for this wonder spice (Poudyal *et al.* 2023*a*). The increase in annual national production and adoption by food manufacturing companies suggests that Akabare chili has promising market potential. It could also be a good source of biocapsaicin (capsaicin extracted from chilies) with pharmaceutical scope (Yasin *et al.* 2023). Bacon *et al.* (2017) have reported potent antimicrobial activity in chili extract, especially against *Listeria monocytogenes* and gram-positive bacteria. Chili as a natural ingredient has the potential to improve the sensory qualities, shelf life and nutritional value of various products in the food, pharmaceutical and cosmetic industries (Anunciato and da Rocha Filho 2012; Andersen *et al.* 2017; Baenas *et al.* 2019). Chili fruit is a good source of micronutrients including carotenoids, capsaicin, crude protein, carbohydrates, ascorbic acid, crude fibre, calcium, potassium and sodium (Litoriya *et al.* 2014; Olatunji and Afolayan 2018).

The changing climate can bring severe heat and drought waves to the horticultural fields, resulting in reduced plant productivity. Between 1850–1900 and 2011–2020, the global average surface temperature increased by 1.09 °C, with a more notable rise over land (1.59 °C) than over the ocean (0.88 °C) and projections anticipate further increases of 1.5 °C by 2040 and 2 °C by 2050 (IPCC 2023). In the Hindu Kush Himalayan region including Nepal, where agriculture is a cornerstone of the livelihoods, the sector has a climate-sensitive ecosystem and is exceptionally vulnerable to global warming and climate change (Sellmyer 2017; Karn and Sharma 2021). Despite an increase of 53 % in hectarage and 27.7 % in total production in 2021, the productivity of Akabare chili decreased by 16.5 % compared to the previous year (MoALD 2022). Such reduction in the production of cash crops could be due to climatic shifts including a rise in annual temperature and altered precipitation (Ranjitkar *et al.* 2016; Karn and Sharma 2021).

Crop plants respond to elevated temperatures and limited soil moisture by making significant modifications in physiology (Aprile *et al.* 2013). Heat and drought stress affect major physiological processes in plants, including the maximum quantum yield of photosystem II (PS II), net photosynthetic rate (*P*_N_) and stomatal conductance (*g*_*s*_) (Baker and Rosenqvist 2004). Drought affects biomass accumulation, root-to-shoot ratio, photosynthesis and transpiration in chili (Phimchan *et al.* 2012; Zamljen *et al.* 2020). Similarly, heat stress reduces the yield performance of chili due to decreased efficiency in gas exchange and photosynthesis (Rajametov *et al.* 2021). Both drought and heat stress inhibit the growth and development of *Capsicum* species by reducing cell proliferation and expansion, leading to a reduction in aerial and root biomass (Poudyal *et al.* 2017; Guo *et al.* 2020; Gisbert-Mullor *et al.* 2023). Heat stress in the open field is often associated with, and induced drought stress (Ahuja *et al.* 2010). Plants’ responses to combined stress result in unique physiological, transcriptional and metabolic effects that differ from those of individual stresses (Zhou *et al.* 2017*b*; Comas *et al.* 2019; Abdelhakim *et al.* 2022).

Chili is a summer crop that commonly experiences drought and heat stress individually or in combination, leading to reduced field performance and yield throughout its ontogeny (Ruiz-Lau *et al.* 2011; Zamljen *et al.* 2020; Wassie *et al.* 2023). Stress also changes the level of leaf pigment compositions, for example, a decrease in chlorophyll content (up to 77 %) as soil dryness intensifies (Wassie *et al.* 2023). Previous studies in chili have focused primarily on crop responses to single stressors (Pagamas and Nawata 2008; Gajanayake *et al.* 2011; Phimchan *et al.* 2012; Jeeatid *et al.* 2017; Mahmood *et al.* 2021), with limited research on combined stimulants (Genzel *et al.* 2021; Reimer *et al.* 2022). Studying crop's responses not only during stress conditions but also during the post-stress recovery phase will provide valuable insights into understanding stress-tolerant physiological mechanisms (Poudyal *et al.* 2019; Rosenqvist *et al.* 2019).

It is imperative to assess a plant’s capacity to restore the functionality of major physiological processes like chlorophyll *a* fluorescence (*F*_*v*_/*F*_*m*_), *P*_N_, *g*_*s*_ and water use efficiency (WUE) (Reynolds-Henne *et al.* 2010; Tovignan *et al.* 2023). *F*_*v*_/*F*_*m*_ is nowadays considered an indicator of photosystem II efficacy or integrity (Sipka *et al.* 2021). Subsequent study of genotypes under stress treatment and recovery conditions is important to explore and identify landraces that are tolerant to specific stimuli or their combinations under particular growing conditions, thereby expanding the genetic diversity (Yumnam *et al.* 2012; López Castilla *et al.* 2019; Cortés and Barnaby 2023; Donoso and Salazar 2023). This adaptive strategy serves to safeguard horticultural crop production (Rijal *et al.* 2022). Genetic diversity present in chili landraces (Castillo-Aguilar *et al.* 2021; Zou and Zou 2021; Cortés and Barnaby 2023; Guo *et al.* 2023) can substantially enrich the genetic foundation of breeding materials. A higher genetic diversity among chili landraces enhances the gene pool by incorporating a wider array of traits, thereby bolstering adaptability, resilience, disease resistance, culinary attributes and innovation potential, all of which ultimately benefit farmers through diversified and sustainable productivity.

Despite the market potential of Akabare chili, activities towards crop improvement and variety development are very scanty. Most of the disciplinary areas of this crop remained untouched. Specifically, studies examining the physiological responses of Akabare chili landraces to either individual stressors or their combined effects, such as drought and heat, represent a novel advancement in the field of phenomics. The study postulated that Akabare chili landraces share physiological responses under drought and heat stress with common chili and other crops. It focused on physiological traits like *F*_*v*_/*F*_*m*_, *P*_N_, *g*_*s*_, stomata anatomy and leaf cooling (LC), while identifying factors influencing total biomass production. Additionally, it assessed post-stress recovery of *F*_*v*_/*F*_*m*_, gas exchange behaviour, stomata anatomy and leaf water status and compared Akabare chili landraces under control, heat, drought and combined stress. Finally, the study discussed the results in the context of understanding the stress-tolerant mechanisms of Akabare chili and put forward potential stress-tolerant landraces for future plant breeding initiatives.

## Materials and Methods

### Seedling preparation and experimental setup

In 2019 and 2020, observational trials were conducted to evaluate 28 Akabare chili landraces from Nepal for their homogeneity. The trials were conducted in Thankot, Kathmandu (27.688235°N, 85.221221°E, 1470 m above sea level) and Salyan (28.336786°N. 82.231586°E, 954 m above sea level). Finally, eight Akabare chili landraces were chosen based on similar phenology and their seeds were bulked. Further information on the pre-evaluation trials and plant materials used are detailed in the supplementary materials [see Supporting Information—Table S1] under the sub-heading ‘Development and pre-evaluation of plant materials’.

This study used nine chili genotypes, which included eight Akabare chili landraces and one commercial cultivar, ‘Jwala’ obtained from SEAN Seed Service Center Ltd., Kathmandu. Jwala is a commercial chili variety in Nepal and was used as a standard check. Among the eight landraces, five, namely, C44, C45C, C62, C64B and C64C, were sourced from the National Agriculture Genetic Resources Center (Gene bank), while the remaining three, BJ77, DKT77 and PPR77, were collected from Ilam, Dhankuta and Panchthar districts of Nepal, respectively.

On 9 August 2021, nine chili genotypes were sown in plastic trays. Each seed was planted separately in a plug tray with a sphagnum substrate (Pindstrup 2, Pindstrup Mosebrug, Ryomgaard, Denmark) in greenhouse no. 3, the Department of Food Science at Aarhus University, Agro Food Park (56.198164°N, 10.155551°E, 48 m above sea level). The seedlings were transplanted into plastic pots (11 cm in diameter and 9 cm in height) 25 days after germination. The pots were positioned with a 10 cm gap between and within rows, and the seedlings were cultivated in a greenhouse under long-day conditions (16 h). To supplement natural light, LED fixtures (FL300, Senmatic, Søndersø, Denmark) were used. The greenhouse had a photosynthetic photon flux density (PPFD) range of 300–400 µmol m^−2^ s^−1^ with 60 ± 5 % relative humidity and an average CO_2_ concentration of 340 ppm. During the day, the air temperature was kept at 28 ± 2 °C, and during the night it was kept at 20 ± 2 °C. The seedlings were irrigated by flooding the benches with a nutrient solution every morning for 10 min (EC = 2.4 mS/cm, *N* = 183 ppm, P_2_O_5_ = 34 ppm, K_2_O = 266 ppm, Mg = 41 ppm, Ca = 152 ppm, Fe = 1.173 ppm, Mn = 1.373 ppm, Cu = 0.159 ppm and Zn = 0.229 ppm).

Seedlings were kept at 28/20 °C for 2 days and then at 30/22 °C for 3 days for a duration of 16/8 h day/night with 60% RH, 400 ppm ambient CO_2_ and PPFD of 400 µmol m^−2^ s^−1^ in the walk-in climate chambers PSI Fytoscope FS-WI (Photon Systems Instruments, Drasov, Czech Republic) for seedling hardening and acclimation. Similarly, in the second chamber, another set of seedlings in heat stress condition (HT) and combined drought and heat stress condition (DHT) were preconditioned for 24 h at 32/24 °C before the onset of stress. To avoid any possible confounding effects of VPD variations on the impact of heat and drought stresses, a stable level of relative air humidity was maintained consistently throughout the experiment. An even VPD at 30 °C and 40 °C temperature levels were maintained in the climate chambers equipped with air humidification and an elevated level of air recirculation.

**Figure 1.**
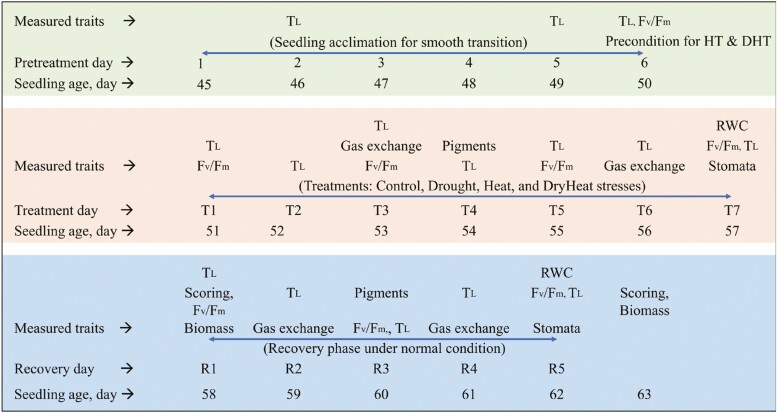
Workflow chart showing the total days of treatment with the corresponding traits measured during the respective treatment. Control, HT: Heat stress treatment, DHT: Combined drought and heat stress treatment (DHT or DryHeat). Forty-five-day seedlings were used in the experiment. Measured traits: *F*_*v*_/*F*_*m*_, chlorophyll a fluorescence as the maximum quantum yield of PS II; gas exchange: net photosynthetic rate (*P*_N_), stomatal conductance (*g*_s_), transpiration rate (*E*), electron transfer rate (ETR), intercellular CO_2_ concentration (*C*_i_); leaf pigments, chlorophyll and flavonol index assessment; RWC, leaf relative water content; Stomatal behaviour including stomata density, length, width, area of stomata (SA) and stomata pore area (PA); *In situ T*_*L*_, leaf temperature; scoring, WS, HII and leaf heat damage. The letters T1 through T7 represent the treatment days, while the letters R1 through R5 represent the recovery days. Contrasting background colours highlight the growing conditions.

The treatments were conducted using 45-day-old seedlings. The experiment consisted of four different treatment conditions: (1) control treatment (CT), which maintained a temperature of 30/22 °C (day/night) and was well irrigated, (2) heat stress treatment (HT), which subjected the seedlings to a temperature of 40/32 °C (day/night) and was well irrigated, (3) drought stress treatment (DT), which was maintained at 30/22 °C (day/night) and was not irrigated and (4) combined drought and heat stress treatment (DHT or DryHeat where meaningful), which subjected the seedlings to a temperature of 40/32 °C (day/night) and was not irrigated throughout the treatment period. All treatments were conducted under 400 ± 10 ppm ambient CO_2_, 60 ± 2.5 % RH and 400 ± 10 µmol m^−2^ s^−1^ PPFD. Seedlings were arranged in a completely randomized design. Among the four sets of 54 seedlings, one set was assigned to control conditions, the other set was subjected to drought stress, spread across two benches each in the same chamber. Similarly, a matching set of seedlings underwent heat stress, and a combination of heat and drought stress across two benches each in another chamber, with six biological replicates per genotype. The irrigation in the drought stress plot was stopped 4 h before the onset of thermal stress in another chamber to make both stresses occur simultaneously. To synchronize the measurements at the beginning of the photoperiod in both chambers, a 2 h time difference was set between the photoperiod of the two chambers. The stress conditions lasted for 7 days, as some seedlings under DHT showed high leaf wilting and scorching symptoms and reached the 75 % threshold for foliage wilting on day 7 (Fig. 1). In CT and HT, and during recovery, seedlings were irrigated with a nutrient solution (pH 5.8 and EC 2.08 mS cm^–1^) twice a day for 10 min, flooding the benches to ensure adequate hydration. The recovery phase started after the end of stress conditions and seedlings were continued for 5 days under control conditions (CR), heat stress recovery (HR), drought stress recovery (DR) and combined stress recovery (DHR) conditions. There were 24 seedlings per genotype. The details about the type of treatment and measured traits on specific days with seedling age are illustrated in Fig. 1.

### Assessment of morphophysiological characteristics

The chlorophyll *a* fluorescence (*F*_*v*_/*F*_*m*_) was assessed in pre-treatment (PT), treatment day (T1, T3, T5, T7) and the recovery phase (R1, R3 and R5) using a MINI-PAM fluorometer (Heinz Walz GmbH, Effeltrich, Germany). The fully developed and light-exposed leaves were selected from the outer and upper canopy, excluding the midrib. Before taking measurements, the adaxial surface of the leaves was dark-adapted for 30 min using a clip (DLC-8, Heinz Walz GmbH) and *F*_*v*_/*F*_*m*_ was measured as the maximum photochemical efficiency of PS II. The measurements were made each day between 12:00 h and 14:00 h and an average of six observations per genotype was considered as the final reading. To provide sufficient time for measurements, one of the two chambers was programmed with a 2 h delay in the day–night cycle. Four observations of *F*_*v*_/*F*_*m*_ per genotype per treatment were taken and averaged for further calculation.

To measure the leaf temperature (*T*_L_) for each genotype under control, stress and recovery conditions, *in situ T*_L_ was recorded throughout the experiment days in three replications using a Raynger 3i infrared gun (Raytek, Santa Cruz, CA, USA) during the daytime 12:00 h, 14:00 h and 17:00 h and these values were averaged to calculate a value per day. The *T*_L_ value obtained for a specific genotype was used as the reference *T*_L_ for gas exchange measurements for the corresponding treatment conditions and used to calculate temperature depression according to Balota *et al.* (2008) and redefined as leaf temperature depression (LTD) since fully expanded leaves from uppermost canopy were used while measuring *T*_L_. Ambient air temperature (*T*_A_) was used as the reference temperature in all stress treatment and recovery conditions. The LTD (°C) was calculated as $\text{LTD=[leaf temmperature }(T_{\text{L}})-\text{ambient temperature }(T_{\text{A}})]$ Similarly, the leaf cooling (LC) percentage was calculated as $\%\text{LC}=[T_{\text{A}}-T_{\text{L}}]\times 100\%$. *T*_L_ was measured at the same height of the canopy all the time and *T*_A_ was obtained from the chamber control unit.

Seedlings were evaluated for stress injury based on the morphological symptoms at the end of stress treatment. The seedlings under high temperatures (HT and DHT) were evaluated following the method of Hong *et al.* (2009) with modifications. The heat injury index (HII) was determined using a five-stage scale, where (i) no macroscopic thermal injury, (ii) moderately dehydrated seedlings with crinkled lower and middle leaflets, (iii) severely dehydrated seedlings with crinkled upper leaflets, (iv) seedlings with many withered leaves and (v) dead seedlings. Similarly, seedlings under DT and DHT were evaluated for the wilting score (WS) according to a five-stage scale. A score of 1 was given for fully turgid seedlings, 2 for slightly wilted seedlings, 3 for moderately wilted seedlings, 4 for highly wilted seedlings and 5 for flaccid seedlings. Both scoring assessments were carried out in an unbiased manner, 1 h after the measurement of *F*_*v*_/*F*_*m*_ and before any destructive measurements were performed.

*P*_N_, intercellular CO_2_ concentration (C*i*), *g*_*s*_, transpiration rate (*E*) and electron transfer rate (ETR) were measured *in situ* using a portable open-system infrared gas analyzer (CIRAS-2, PP Systems, Amesbury, MA, USA) in the climate chamber. A gas exchange measurement was taken four times during the experiment, two during the stress condition (T3 and T6) and two during the recovery phase (R2 and R4) with three plants per genotype. Measurements were taken at a 400 µmol m^−2^ s^−1^ PPFD provided by the CIRAS-2 LED light source. The reference level of CO_2_ concentration (C_*r*_) was set at 400 ppm. The leaf temperature in the cuvette was set according to the daily average *in situ* leaf temperature recorded previously as 20 °C, 32 °C, 23 °C, 34 °C, 22 °C, 24 °C and 25 °C for CT, HT, DT, DHT, HR, DR and DHR, respectively. The setting values in CIRAS-2 for CO_2_ level, cuvette temperature and light intensity during the measurement were meant to align with the specific growth conditions in each treatment. The first fully expanded leaf was placed in a 2.5 cm^2^ cuvette and the results were captured every 10 s after a *P*_N_ and *g*_*s*_ stabilization period of 10–25 min. The average of the last 10 measurements was considered as the result. During the gas exchange measurement, the vapor pressure deficit was kept below 2.0 kPa. The instantaneous WUE was calculated as the ratio of *P*_N_ and *E*, $\left[\text{WUE}=(P_{N}/E)\right]$.

The chlorophyll content index (CCI) and flavonol content index (FCI) within the leaves (area 9.5 mm in diameter) were non-destructively assessed using the MPM-100 (Opti Sciences, Inc., Winn Avenue, Hudson, NH), a portable field instrument for measuring pigment levels via optical transmittance and fluorescence properties of the leaf. Pigments were measured twice a day (11:00 and 14:00 h) on treatment day 6 (T6) and recovery day 4 (R4) in the uppermost matured leaves, in four biological replicates per genotype per treatment. The result per genotype per treatment was an average of eight readings each day.

To measure the leaf relative water content (RWC) on treatment day 7 (T7) and recovery day 5 (R5), the approach described by Smart and Bingham (1974) was followed. Three discs per genotype were cut from young fully expanded leaves, each with a size of 10 mm and then sealed in metallic foil and transported to the laboratory. The discs were weighed using an analytical weighing scale to find their fresh weight (FW) and then floated in distilled water in a Petri dish at room temperature (approximately 16 °C) without light for 12 h to obtain the turgid weight. The discs were dried at 80 °C overnight and weighed for the dry weight (DW). Finally, the leaf RWC was calculated as RWC=(FW−DW/TW−DW)×100%.

A red string was used to tag the stem just below the petiole of unfolded leaves (<2 cm × 1 cm dimension) of each plant after the PT. On treatment day 7 (T7) and recovery day 5 (R5), stomatal impressions were captured from the abaxial side of the first fully expanded and tagged leaf using the silicon rubber impression technique, aimed at assessing stomata size and density. Impressions were taken in three biological replicates per treatment, excluding the midrib, using elite HD + (Zhermack, Badia Polesine, Italy) on T7 and R5. The impressions were taken 2 h after the onset of the light period to allow dark-exposed seedlings to reach steady-state operating stomatal conductance. Thin layers of nail varnish prepared on the original imprints were used in the microscopic slides and pictures were taken from three non-overlapping fields using a Nikon AZ100 microscope (Nikon Corp., Tokyo, Japan) mounted with a Nikon DS-Fi1-U2 camera. Labelled images were then analyzed using ImageJ software (ImageJ, Version 1.53p, National Institute of Health, USA) as a tool for biological image study (Schindelin *et al.* 2012) to estimate the field of view, the number of stomata, stomata length (SL), stomata width (SW), stomata pore length (PL) and pore width (PW). Stomata density (SD) was determined based on the total number of stomata per area of field of view, avoiding interveinal areas. We assumed an elliptical shape of the stomata pore and a rectangular shape of the stomata while calculating respective areas. Additionally, we calculated the ratio between the pore area (PA) and stomata area (SA) as PASA = (PA/SA). Genotypes C64C and PPR77 were excluded from the data analysis since a few leaves (1 and 2 leaves, respectively) were developed during the combined drought and heat stress treatment.

At the end of the stress conditions, three seedlings of each genotype were harvested under the conditions of CT, DT, HT and DHT. Similarly, after the completion of the recovery phase (R5), the remaining three seedlings per genotype were harvested for fresh and DWs. The seedlings were cut at the cotyledonary node and the fresh leaves were removed to measure the FW and the leaf area (LA). The leaf was counted fully unfolded and had a minimum leaf blade dimension of 1 cm × 2 cm (without petiole) and considered for leaf measurements. LA was measured using a LA metre (Model 3100, LI-COR Inc., Nebraska, USA). Fresh shoot weights were recorded on the same day of harvesting, and root weights were obtained the following day after carefully washing the roots with water and draining the water from the root surface.

Total vegetative fresh weight (TVFW) was obtained by adding FWs for leaf, stem and roots. The DWs of the seedling parts, including the leaf DW (LDW), stem dry weight (SDW) and root dry weight (RDW), were determined by drying the fresh mass of each part in an oven at 70 °C for 72 h. The total shoot dry weight (ShDW) was calculated as the sum of the LDW and SDW. Similarly, total vegetative dry weight (TVDW) was obtained by adding RDW to the ShDW. The leaf mass per leaf area (LMA) was calculated by dividing the LDW by the leaf area (LA), and the root-to-shoot ratio (RS) was obtained by dividing the RDW by the ShDW. The dry matter proportion (DMP) of each genotype was calculated as a ratio of TVFW and TVDW of the respective genotypes and expressed in percentage, DMP = (TVDW/TVFW)×100%.

### Data analysis

The study recorded a total of 40 variables and 10 derivatives during the experiment. For data analysis, only 28 specific traits were considered. Data of biomass measurement, stomata anatomy, stress scores and leaf pigments were subjected to ANOVA with three factors (genotype, treatment and stress condition). ANOVA models showing significant interaction between genotypes and treatment conditions were further entered into the pairwise *t*-test. Data sets of chlorophyll *a* fluorescence, gas exchange measurements and leaf temperature were undergone through repeated measures ANOVA to analyze the changes in mean values over different time points and conditions. The difference in plants’ performance among genotypes under different four treatment conditions was analyzed by comparing mean values using Tukey’s HSD as a post hoc test at a significance level of *α* = 0.05 to observe the existing variation in genotypes and treatments for the measured traits. Stress and recovery values were compared to control values for each treatment level, mean values for individual genotypes were compared with standard check (Jwala) and the data were presented as mean ± standard deviation (*SD*). The Pearson correlation coefficient was calculated to assess the linear relationships among variables under control, stress treatments and recovery conditions. Bivariate correlation was done to reveal significance in correlation among the measured parameters under three conditions separately. Multiple linear regression was employed to investigate the predictors of total biomass production. Data were analyzed using SPSS (IBM Corp. Released 2011. IBM SPSS Statistics for Windows, Version 20.0. Armonk, NY: IBM Corp) and results were visualized using SigmaPlot for Windows Version 15.0.0.13 (Inpixon HQ, Palo Alto, CA).

## Results

**Figure 2.**
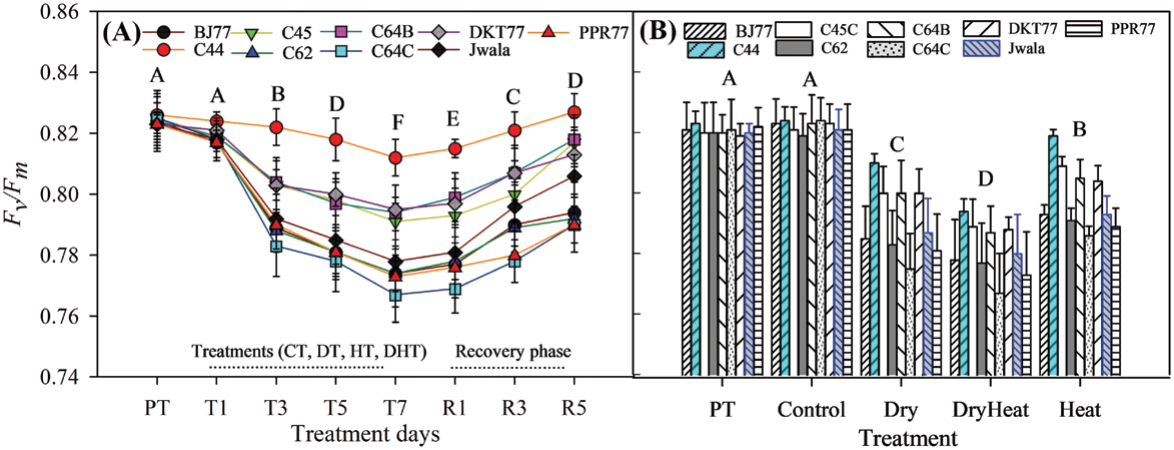
The chlorophyll *a* fluorescence (*F*_*v*_/*F*_*m*_) during the stress treatments and recovery phase. (A) *F*_*v*_/*F*_*m*_ values averaged for PT, control (CT), drought (DT), heat (HT) and a combination of drought and heat stresses (DHT) for different treatment days (T1–T7) and the recovery phase (R1–R5). Stress treatment started on day T1 (treatment day 1) under four conditions, CT, DT, HT and DHT, and lasted for 7 days. A five-day recovery started from R1 (recovery phase, day 1) under normal conditions. Labels above the *X*-axis coincide with treatment days showing different treatment conditions. (B) *F*_*v*_/*F*_*m*_ values averaged for different treatment conditions. Data are mean ± *SD* (*n* = 6) and different letters above the line and bar graphs denote a significant difference (*P* < 0.05).

Average mean values of *F*_*v*_/*F*_*m*_, *E*, C*i*, *g*_*s*_, *P*_N_ and ETR obtained for the measurement day differed significantly. The *F*_*v*_/*F*_*m*_ values of Akabare chili landraces showed significant variations (*P* < 0.05) under different growing conditions (Fig. 2). Under control conditions, the average *F*_*v*_/*F*_*m*_ of the nine genotypes was 0.813. This value decreased by 2.95 % under HT, 3.63 % under DT and 4.92 % under DHT. All genotypes showed decreased *F*_*v*_/*F*_*m*_ values under stress. Among the landraces, C44, C45C, C64B and DKT77 exhibited significantly higher *F*_*v*_/*F*_*m*_ (>0.807) under stress conditions, while the remaining five (BJ77, C62, C64C, Jwala and PPR77) showed lower *F*_*v*_/*F*_*m*_ (<0.795) (Fig. 2A and B). During the recovery of corresponding stresses (R1–R5), average *F*_*v*_/*F*_*m*_ increased by 1.96 ± 0.005 % across the genotypes. The C44 exhibited the highest values (average 0.821, max 0.832) across all stress treatments, and C64C showed the lowest values (average 0.785, min 0.771). The *F*_*v*_/*F*_*m*_ value of C44 during the recovery phase (R5) did not differ from the PT value (Fig. 2A). The average values of *F*_*v*_/*F*_*m*_ in the PT and control were significantly higher than any stress treatment, HT showed significantly higher values of *F*_*v*_/*F*_*m*_ than DT and DHT and the significantly lowest values of *F*_*v*_/*F*_*m*_ recorded under DHT for all genotypes (Fig. 2B).

**Figure 3.**
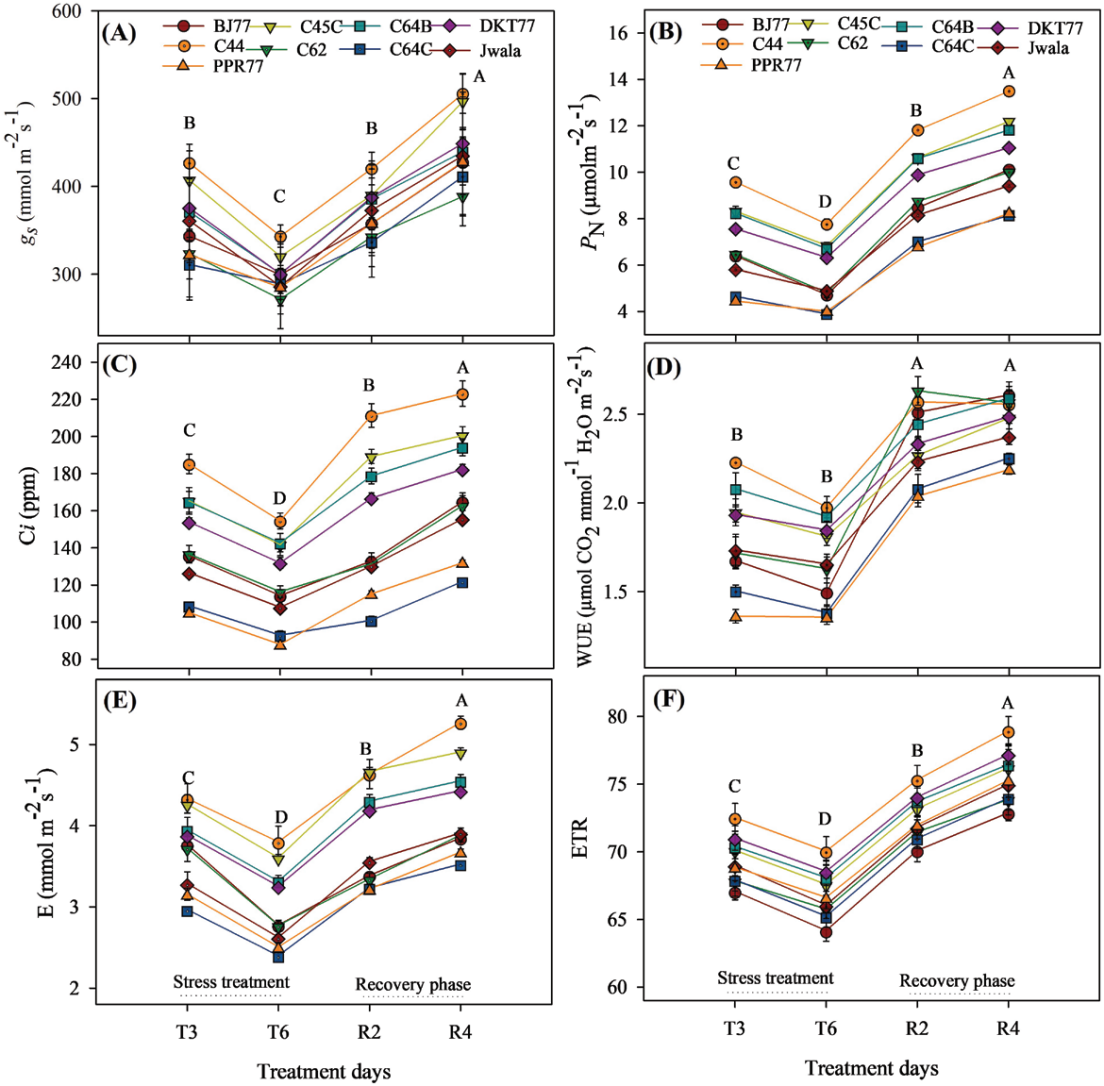
Photosynthetic gas exchange parameters of eight Akabare chili landraces and Jwala under heat, drought and combined stresses were taken in the treatment days and recovery phase. Treatment starts on day 1 (T1) and ends on day 7 (T7) followed by a 5-day long recovery phase (R1 to R5). (A) gas exchange (*g*_*s*_); (B) net photosynthetic rate (*P*_N_); (C) intercellular CO_2_ concentration (C_*i*_); (D) WUE as a ratio of *P*_N_ to *E* (*P*_N_/*E*); (E) transpiration rate (*E*) and (F) ETR measured in the first fully expanded leaves during different treatments. Data are mean values ± *SD* (*n* = 3) and different letters show significant differences (*P* < 0.05).

The simultaneous occurrence of heat and drought stress had a significant impact on various gas exchange parameters such as *P*_N_, *g*_*s*_, C*i*, *E* and ETR resulting in a different level of WUE (Fig. 3). Among landraces, C44, C45C and C64B exhibited stronger physiological responses for *P*_N_, *g*_*s*_, C*i*, *E*, WUE and ETR, while such responses for C64C, Jwala and PPR77 were significantly lower.

The stomatal conductance decreased significantly under stress treatments (Fig. 3A). Compared to the control value, *g*_*s*_ was decreased by 8.7 % under heat, 14.6 % under drought and 30.7 % under DHT stresses. The highest *g*_*s*_ was recorded on R4 and the lowest was recorded on T6. Landraces C44 and C45C showed higher *g*_*s*_ values throughout the measurement days, while C62 and C64C had significantly lower values under all treatment conditions and the other five genotypes remained in between.

The net photosynthetic rate decreased significantly under stress conditions (average 39.9 ± 2.7% less than the control value) (Fig. 3B). The average *P*_N_ for R4 was significantly the highest, followed by R2, while T6 produced the lowest *P*_N_ value. For landraces, C44 showed the highest *P*_N_ values both in the treatment and recovery phase and PPR77 and C64C showed the significantly lowest net photosynthetic rate across the treatment days.

The intercellular CO_2_ concentration showed a similar pattern to *P*_N_. In R4, C*i* was significantly highest among the measurement days (Fig. 3C). The mean C*i* recorded in T6 was the lowest and values for T3 and R2 were statistically different. Among the landraces, C44 and C45C exhibited significantly higher C*i* values compared to other genotypes, while C64C and PPR77 were ranked at the lower end.

Higher values for WUE for all genotypes were observed during the recovery phase compared to stress conditions (Fig. 3D). Compared to normal values, on average across the three treatments, WUE was reduced by 25.2 % under stress conditions. The highest reduction was seen under DHT (34.9 %), followed by DT (21.4 %) and HT (19.2 %). Significantly the highest values of *E* (Fig. 3E) under R4 were recorded, followed by R2. T6 significantly produced the lowest *E* values among the treatment days. Landrace C44 showed significantly higher values among the genotypes. Regarding ETR (Fig. 3F), R2, T3 and T6 displayed values that were 4 %, 8.1 % and 11.4 % lower than R4, respectively. The most significantly elevated values were observed for R4, while T6 exhibited the lowest values among all. Notably, among the genotypes, C44 displayed the highest ETR values significantly, while BJ77 recorded the lowest values (Fig. 3F).

**Figure 4.**
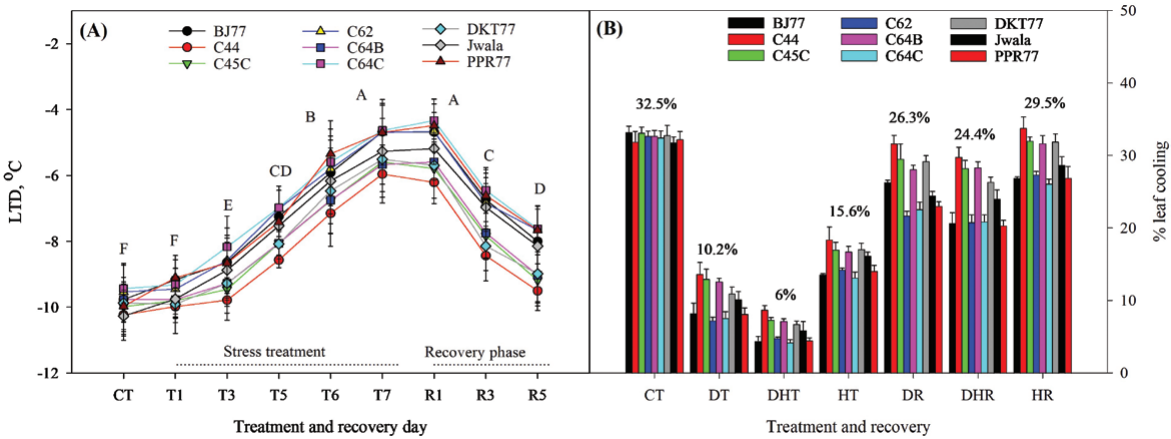
Leaf temperature depression and percent leaf cooling under stress treatment and recovery phase. (A) LTD (°C) calculated as (leaf temperature, *T*_L_ - ambient temperature, *T*_A_) of eight Akabare chili landraces and Jwala measured during control treatment (CT), stress treatments (heat, drought and combined of both stresses from T1 to T7) and during the recovery phase (from R1 to R5). (B) Percentage of leaf cooling (% LC) calculated as [(*T*_A_ - *T*_L_) ÷ *T*_A_] × 100 % under different treatment conditions in, T7 and recovery phase, R5. Data are mean values ± SD (*n* = 12) and different letters show significant differences (*P* < 0.05). The values above the bars are the average % leaf cooling for corresponding conditions.

On average, leaf temperature was 7.6 °C cooler than ambient air temperatures with almost 24 % of LC across the treatments (Fig. 4A and B). The average values of LTD under different treatment and recovery conditions ranged from −4.3 °C to −10.5 °C, while LC ranged from 12 % to 35 %. The leaves of C44 were found to be significantly cooler than the other eight genotypes during the stress treatment and recovery phase. A significantly lower LC (warmer leaf) was seen for T6, T7 and R1 compared to T3, T5 and R5. The coolest leaves were observed for PT and T1, followed by T3 (Fig. 4A). The combined stress of drought and heat exhibited the significantly lowest LC percentage at 6 %, followed by 10.2 % for drought stress and 15.6 % for heat stress (Fig. 4B). DHR showed significantly warmer leaves compared to DR and HR. However, plants under HR fully recovered LC, like those under CT.

**Figure 5.**
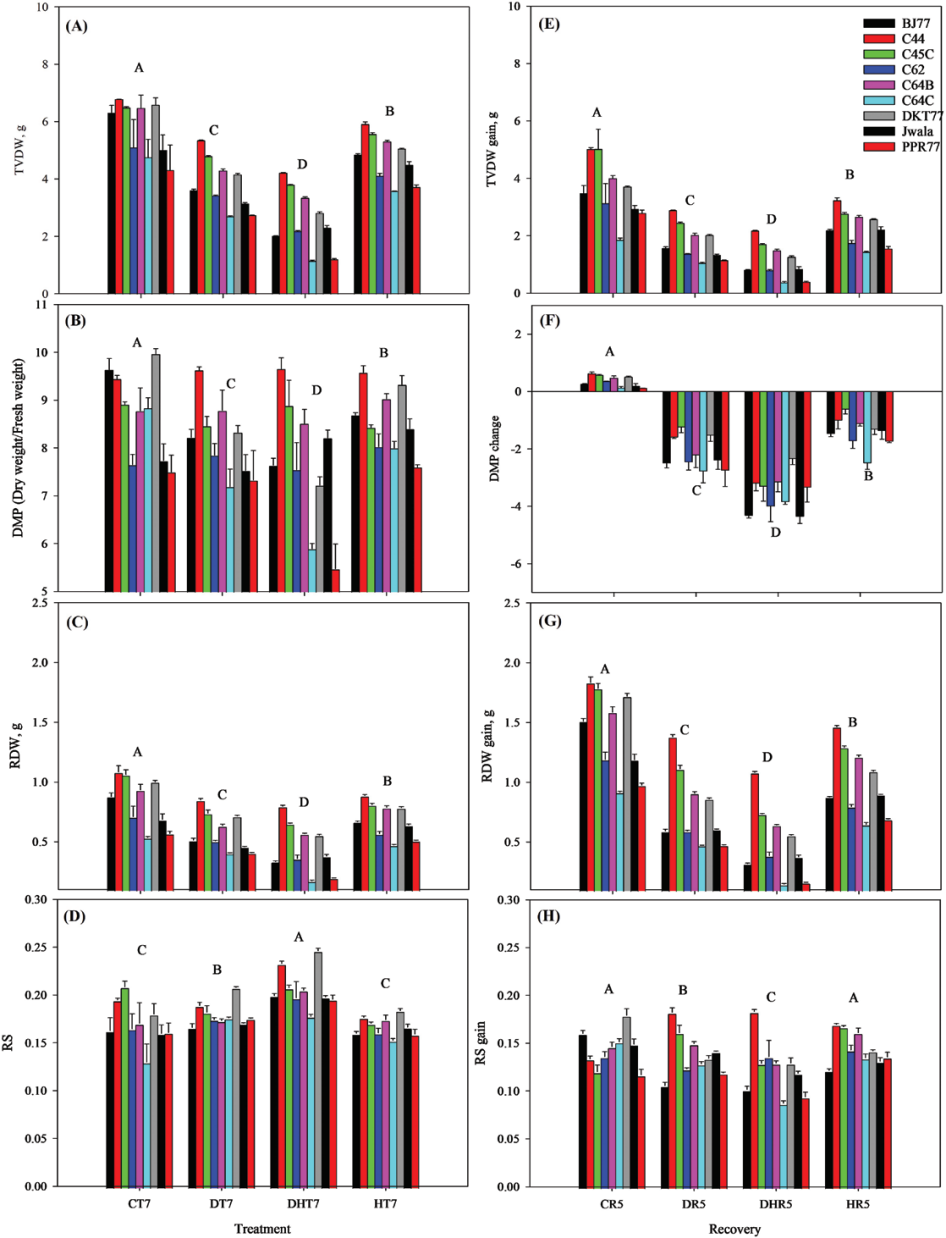
Production and partitioning of dry biomass of chili genotypes under treatment and recovery phase. The measurement was done at the end of treatment, treatment day 7 (T7) and the recovery phase, recovery day 5 (R5). Gain in respective biomass during the recovery is presented in the right column. (A) total vegetative dry weight (TVDW), g; (B) dry matter proportion (DMP), %; (C) root dry weight (RDW), g and (D) root-to-shoot ratio; (E) – (H) shows the gain in corresponding biomass during the recovery phase of associated treatment conditions. CT denotes control, DT drought stress, HT heat stress, DHT denotes combined drought and heat stress conditions on treatment day 7, and CR, DR, HR and DHR denote recovery of respective treatments on recovery day 5. Data are mean values ± *SD* (*n* = 3). Different letters represent significant differences (*P* < 0.05) and the figures above the bar diagram are average values of corresponding conditions.

Genotypes performed differently for total biomass production under different treatment conditions and recovery phase (Fig. 5). There was a 57.5 % reduction in TVDW due to combined stress followed by drought (37.8 %) and heat (21.4 %) compared to control (Fig. 5A). The gain in TVDW under the recovery was significantly higher in CR (35.2 %), followed by HR (32 %), DR (31.1 %) and DHR where DHR showed the lowest gain percentage (28.7 %) (Fig. 5E). Average across the treatment recoveries (HR, DR, DHR), C44 gained a significantly higher amount of TVDW (35 %), followed by DKT77 (33.48 %) and C45C (33 %) and the lowest recovery in TVDW was observed for C64C and PPR77 (29 %). The lowest value of DMP was observed in DHT7, 19 % lower than CT7 during stress conditions (Fig. 5B). The value of DMP in DHR5 was also 22 % lower than that of CR5 during the recovery phase. The highest DMP values were obtained for all genotypes in CR5, a control condition that continued during the recovery phase, followed by HR5, DR5 and CT7. Compared to the control, gain in DMP under the recovery phase was found negative and exceptionally low in DHR (−100.7 %) (Fig. 5F). RDW was severely reduced by 47 % under DHT, 30 % under DT and only 18 % under HT (Fig. 5C). Plants under the recovery phase recovered a significant amount of root biomass (50.5 %–63 %), whereas landraces C44, C45C, C64B and Jwala gained more than 57 % RDW. Similarly, the highest value of RS was found in DHR5, followed by DR5 and HR5 (Fig. 5D). Under stress, the highest RS was noticed for DHT7, 30.8 % higher than the control condition. DT7 and HT7 produced 13.6 % and 5.8 % higher RS compared to CT7, respectively. Gain percent in RS varied from 37 % to 49.7 %, the highest in control followed by HR (46.4 %), DR (43.3 %) and the lowest in DHR. Landrace C44 showed a significant gain in RS (47.88 %) compared to others and the lowest was recorded for PPR77 (42.3 %) (Fig. 5H). Landrace C44 performed well in biomass production and partitioning, and significantly gained a larger proportion of biomass under recovery followed by C45C and DKT77, while C64C, PPR77 and C62 were the bottom performers among the nine.

The results showed that stress conditions had a significant impact on TVFW, ShDW, LDW and LMA, resulting in reductions compared to the control condition and recovery phase [see Supporting Information—Fig. S1]. Detailed results for TVFW, ShDW, LDW and LMA can be found in the supplementary materials under the subheading ‘Biomass accumulation under the stress conditions and recovery phase’.

Under stress conditions, Jwala, PPR77, C62 and C64C had significantly higher WS values, whereas the lowest WS value was observed for C44 [see Supporting Information—Table S2]. Additionally, compared to the other six landraces and Jwala, C44 and C45C had significantly lower HII values. The variation in WS and HII recovery among the genotypes was apparent during the recovery phase. Significantly, the highest values for CCI and relative water content and the lowest values of FCI were observed for stress-tolerant landraces, C44, C45C, C64B and DKT77 [see Supporting Information—Fig. S2]. Detailed results for the leaf relative water content, FCI and CCI can be found in the supplementary materials under the subheading ‘Stress condition increases flavonol index and decreases leaf water content and chlorophyll content index’.

**Figure 6.**
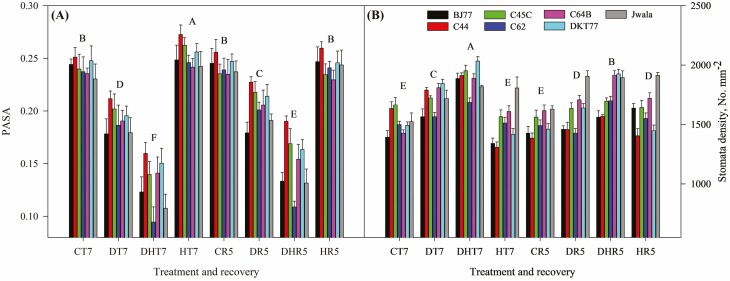
Stomatal anatomy of eight Akabare chili landraces and Jwala varieties grown under four treatment conditions and stress recovery phase. Imprints were independently obtained for the stomatal anatomy on stress treatment day 7 (T7) of the respective treatment and recovery phase day 5 (R5). (A) ratio of pore area (PA) to stomata area (SA), PASA and (B) Stomata density as the number of stomata per mm^2^. CT: control, DT: drought stress, HT: heat stress, DHT: combined drought and heat stress; CR, DR, HR and DHR denote the recovery phase of respective treatments. Data are mean ± *SD* (*n* = 10) and the different letters above the bars show significant differences (*P* < 0.05).

Seedlings under heat stress showed significantly larger PASA compared to other growing conditions (4.6 % larger than control) (Fig. 6A). Compared with the control (CT7), drought and combined stress reduced the PASA by 20.5 % and 46.6 %, respectively. During recovery observations, no differences were found between the average PASA for control and heat recovery. Compared to CR5, significantly lower values were recorded for DR5 and DHR5, by 16.5 % and 42.4 %, respectively. The largest PASA was observed across all treatments for landrace C44, while C64C and PPR77 showed the smallest PASA among the studied genotypes. The number of stomata per square millimeter area was significantly the highest for the DHT7 (1889 ± 119). Stress treatments, except heat stress, significantly increased the SD, 26.3 % by DHT7 and 13.4 % by DT7 (Fig. 6B). For the genotype, Jwala produced significantly the highest SD (1778 ± 154) among 9 across the treatments, which was followed by C64B (3.6 % less than Jwala) and BJ77 produced the lowest SD (13.6 % less than Jwala). Throughout the stress recovery phase, 9.7 % more stomata were observed in stress-recovered seedlings compared to those continuously growing under normal conditions (CR5). SD under HR5, DR5 and DHR5 were significantly higher by 6.9 %, 4.9 % and 17.5 %, respectively, compared to the SD observed for the seedlings in CR5 (Fig. 6B). Detailed results of SW, SL, stomata PW and length are presented as Supporting Information—Fig. S3 in supplementary materials under the subheading ‘Variation in stomata size, pore size, and stomata density’. Similarly, chili plants under different treatment conditions [see Supporting Information—Fig. S4] and during the recovery phase [see Supporting Information—Fig. S5] are illustrated in the supplementary materials of this study.

**Figure 7.**
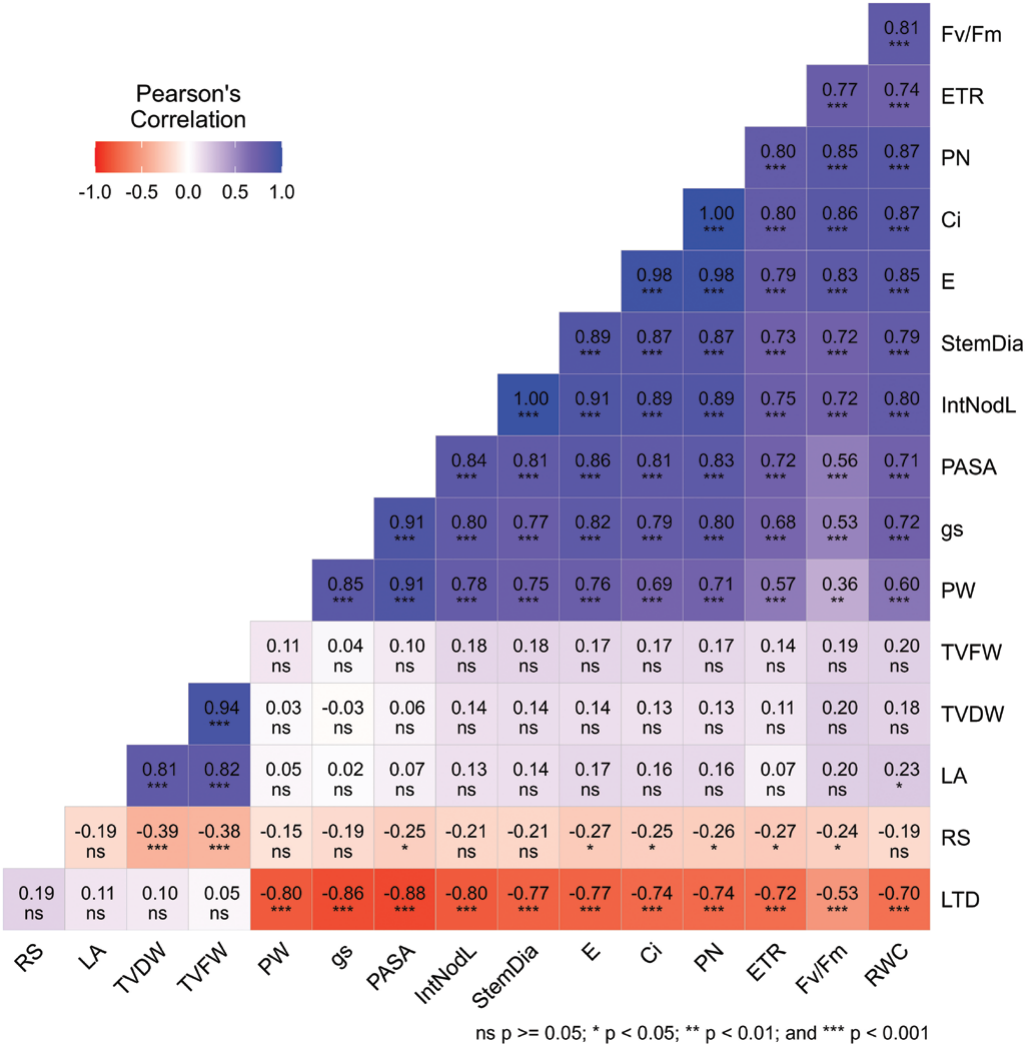
A correlation matrix plot illustrating the interrelationships between 16 morphological and physiological traits observed under stress (average of drought, heat and combined of both stresses) condition. RS, root-to-shoot DW ratio; LA, leaf area; TVDW, total vegetative DW; TVFW, total vegetative fresh weight; PW, stomata pore width; *g*_*s*_, stomatal conductance; PASA, a ratio of stomata pore area to stomata area; IntNodL, internode length; StemDia, stem diameter; E, transpiration rate; *C*_i_, intercellular CO_2_ concentration; *P*_N_, net photosynthetic rate; ETR, electron transfer rate; *F*_*v*_/*F*_*m*_, chlorophyll a fluorescence; RWC, relative water content; LTD, leaf temperature depression; ns, nonsignificant; *P ≥ 0.05; **P ≥ 0.01; ***P ≥ 0.001 level.

A Pearson’s correlation coefficient (*r*) was calculated for selected traits to see the nature of relationships among the variables. The correlation matrix showing the correlation coefficients of 16 variables under stress treatment conditions and level of significance at *P* = 0.05 is illustrated in Fig. 7. Positive and significant correlations (*r* = 0.23 − 1.0) were observed among TVDW, TVFW, LA, *F*_*v*_/*F*_*m*_, *P*_*N*_, Ci, E, ETR, stem diameter, internode length, *g*_*s*_, PW, PASA and RWC. Variables RS and LTD produced significant negative correlations (*r* = −0.24 − −0.88). Similar matrices are annexed in Supporting Information— Fig. S6 (16 variables under control condition), Supporting Information— Fig. S7 (16 variables under recovery condition) and Supporting Information—Fig. S8 (24 variables under stress treatment condition) in supplementary materials.

A step-wise multiple linear regression analysis was systematically conducted, utilizing a set of carefully chosen predictors with no multicollinearity. The outcomes of this regression analysis revealed distinct predictor sets for different conditions. Under control conditions, the variables LTD, *T*_L_ and SD emerged as predictors for TVDW (*R* = 0.592, *R*^2^ = 0.350, adjusted *R*^2^ = 0.311, standard error of estimate = 2.267, *n* = 54). In the stress recovery phase, leaf number and WS were identified as significant predictors of TVDW (*R* = 0.76, *R*^2^ = 0.578, adjusted *R*^2^ = 0.567, standard error of estimate = 1.324, *n* = 81). Conversely, under the stress treatment conditions, only LA significantly predicted TVDW (*R* = 0.814, *R*^2^ = 0.663, adjusted *R*^2^ = 0.658, standard error of estimate = 0.824, *n* = 81). Additional details of the best-fit models for each analyzed condition, including test statistics, are provided in Supporting Information—Table S3.

## Discussion

The study delved into the physiological responses of Akabare chili landraces to drought, heat and a combination of both stress conditions during their growth. Various screening methods have been developed to identify heat and drought-tolerant genotypes within the Solanaceae family at different growth stages (Pagamas and Nawata 2008; Xu *et al.* 2017; Arnaoudova *et al.* 2020). The current study primarily focused on the screening of stress-tolerant Akabare chili landraces at their early vegetative growth stage considering the physiological changes due to drought and heat stress, and their combination. Early detection of plants’ resilience to heat and drought stress will assist efficient use of resources in stress tolerance breeding programmes. The range and duration of elevated temperature and drought stress conditions used in this study were sufficient to segregate Akabare chili landraces based on their differential physiological responses.

The results showed that the distinct differentiation of *F*_*v*_/*F*_*m*_ among the nine chili genotypes was accompanied by a clear separation in gas exchange attributes, such as *E*, *g*_*s*_, C*i* and *P*_N_. Akabare chili landraces showing higher *F*_*v*_/*F*_*m*_ values also demonstrated improved net photosynthetic rate and biomass production under stress, which is a desirable trait to improve field performance as discussed by Poudyal *et al.* (2019). The responses of heat-tolerant tomatoes (Zhou *et al.* 2017*b*) and strawberries (Arief *et al.* 2023) with higher values of *F*_*v*_/*F*_*m*_ under stress conditions align with the responses observed in Akabare chili landraces under combined stress conditions. These findings support the hypothesis that the overall performance of seedlings can be affected if drought and heat stress affect a fundamental part of photosynthesis, such as PS II. Furthermore, stress-sensitive genotypes had lower *F*_*v*_/*F*_*m*_ and slower recovery when returned to normal growth conditions, highlighting the genotype-specific recovery ability of PS II. Landraces with higher *F*_*v*_/*F*_*m*_ also exhibited higher LTD, higher percent of transpiration cooling and could have greater thermal stability of the thylakoid membranes and lesser inhibition of PS II. In agreement with this, Rajametov *et al.* (2021) reported a faster recovery rate of photosynthesis for heat-tolerant chili genotypes. Landraces that were sensitive to drought and heat stress showed a slower recovery in their *F*_*v*_/*F*_*m*_ compared to tolerant genotypes even after 5 days of recovery, showing clear differences among tested landraces. In the case of the Akabare chili landraces, subjecting the plants to a temperature of 40/32 °C for 1 week and a 7-day drought stress period was effective in assessing sensitivity to both heat and drought based on *F*_*v*_/*F*_*m*_ measurements. Nonetheless, the moderate effects of heat stress on physiological processes suggest that exploring these traits under even higher temperature conditions could be a topic of interest for further research.

The average values of *F*_*v*_/*F*_*m*_ under drought stress were significantly lower than those under heat stress, showing a higher sensitivity of landraces to water-limiting conditions. Sensitive genotypes may have experienced chronic photo inhibition during stress conditions, resulting in lower *F*_*v*_/*F*_*m*_ values even during the recovery phase. The disparity in *F*_*v*_/*F*_*m*_ levels during recovery between genotypes implies that recuperation from stress involves distinct processes unique to each genotype (Dalal *et al.* 2019; Zhou *et al.* 2020*a*; Abdelhakim *et al.* 2021; Zhang *et al.* 2023). Minor discrepancies in *F*_*v*_/*F*_*m*_  *F*_*v*_/*F*_*m*_ effectively differentiated treated and control seedlings after 5 days of recovery, indicating that *F*_*v*_/*F*_*m*_ could also be a useful physiological marker (Yin *et al.* 2010; Poudyal *et al.* 2019; Arief *et al.* 2023) in Akabare chili phenotyping. Yet, more research is needed to understand the irreversible damage to PS II during extended drought and elevated temperatures, along with its connection to overall carbon assimilation, as highlighted by Feller (2016).

Landraces with higher *P*_N_ showed higher LTD leading to a higher percentage of LC that could have collinearly contributed to the efficient CO_2_ assimilation. Increased *E*, ETR and WUE might contribute to higher *P*_N_ when *P*_N_ is found positively correlated with higher stomatal conductance. *P*_N_ and *E* likely acted synergistically to uphold carbon uptake while reducing water loss, as the relationship was highlighted by the presence of positive and statistically significant correlations between *P*_N_ and WUE. This aligns with previous research that demonstrated increases in WUE, *P*_N_ and *g*_*s*_ in tolerant tomatoes subjected to heat stress (Sato *et al.* 2002; Abdelmageed and Gruda 2009; Zhou *et al.* 2015), and improved yield and leaf metabolism in contemporary wheat genotypes (Brestic *et al.* 2018; Abdelhakim *et al.* 2022). Recently Fukai and Mitchell (2022) have reviewed the correlations between canopy temperature depression and yield and discussed that genotypes displaying larger temperature depression (cooler leaves) hold promise for enhancing carbon gain. However, other factors besides temperature depression might have played roles in stomatal behaviour. Further understanding is recommended on LTD and its potential contributions to carbon gain in Akabare chili landraces.

During drought, heat and combined stress, greener leaves with lower HII and WS were positively highly correlated with *F*_*v*_/*F*_*m*_, *P*_N_, C*i*, *g*_*s*_*, E*, RWC, WUE and TVDW, while significant negative correlations were observed between LTD and HII, *P*_N_, *g*_*s*_*, E*, WUE and TVDW. This shows that greener leaves contributed to improved leaf metabolism and better stomatal activities through the efficient influx of CO_2_ and the efflux of water from leaves, maintaining an efficient source-sink relationship under stress conditions. The current results are in agreement with previous research that found a reduction in HII, *P*_N_, C*i*, *g*_*s*_, *E* along with premature chlorosis in tomatoes under thermal stress (Zhou *et al.* 2015; Poudyal *et al.* 2019), drought stress-induced reduction of chlorophyll content in tomatoes (Zhou *et al.* 2020*b*) and chili (Sahitya *et al.* 2018; Wassie *et al.* 2023). Stay-green and leaf-cooling traits are gaining popularity as physiological markers for breeding heat and drought tolerance (Deva *et al.* 2020). Future studies on Akabare chili landraces should consider incorporating both morphological and physiological leaf traits into investigations.

Most of the measured traits under different conditions were collinear and highly correlated with total biomass that was also observed in wild chili (Osuna-Rodríguez *et al.* 2023). Multiple linear regression with predictors in stressful growing environments proposed a linear model with LA as a significant contributor to total biomass production indicating that larger LA is a crucial factor under drought and heat stress growing conditions. This could be attributed to the unfolded leaf surface in tolerant genotypes that could have harvested more light with lower WSs and or HIIes compared to sensitive genotypes. However, during the stress recovery phase, significant predictor variables were identified as leaf number and WS. Newly developed leaves (6.5 % gain in leaf number compared to stress) in the upper canopy likely contributed to the carbon gain through the efficient recovery of light-harvesting pigments.

The assessment of leaf pigments such as CCI, FCI and leaf water content aimed to supplement the understanding of how Akabare chili landraces respond to stressful growing conditions and facilitate genotype screening. Our results on RWC are consistent with those of Escalante-Magaña *et al.* (2019) in *Capsicum*, and Park *et al.* (2023) in tomato, who reported decreased RWC levels under stress conditions. A lower RWC value may indicate a lower level of osmotic adjustment in stress-sensitive chili genotypes. Decreased levels of CCI and increased levels of FCI under individual and combined stress corresponded to the previous results in *Amaranthus*, hot and sweet pepper and tomato (Bhandari *et al.* 2018; Netshimbupfe *et al.* 2023). We measured CCI and FCI at approximately the midpoint of the stress treatment and recovery phase, which could account for the lack of trend analysis. Additional research is advised to further delve into the metabolomics of Akabare chili landraces under stress conditions.

The stomatal ability to regulate water use is considered the most important adaptation mechanism of plants in a diverse environment (McAdam *et al.* 2021). A significant increase in the density of stomata (SD) was found under individual and combination of drought and heat stress. Similarly, the ratio of pore area to stomata area (PASA) was found to be more sensitive to stress stimuli. A lower number of stomata and a higher ratio of PASA in tolerant genotypes compared to sensitive ones could have contributed to the efficient exchange of CO_2_. Furthermore, a low SD can contribute to lesser evaporative loss, thereby conserving water and maintaining the turgor. Tolerant genotypes need to have dynamic and faster stomata that are sufficient to increase transpiration, thus increasing photosynthesis (Drake *et al.* 2013). Although the study did not measure the rates of stomata opening and closure directly for individual genotypes under stress, genotypes C44, C45C and DKT77 could have efficient stomatal operation as they showed higher transpiration and stomatal conductance. The dimension of the stomata is an important trait to observe during the stress conditions of landraces because the development of stomata physiology is necessary to achieve high *P*_N_ ability under stress and even during the recovery phase (Westbrook and McAdam 2021). Stomata and pore size observed in this study are specific to growing conditions and vary according to genotype. Drought and combined stress significantly reduced PASA by 20.5 % and 46.6 %, respectively. However, under heat stress, a slight increment of PASA by 4.4 % was observed instead of a reduction. This supports the understanding that drought limits stomata openings, while heat stress cues oppositely and promotes opening to cool the leaf, as discussed earlier (Urban *et al.* 2017; Marchin *et al.* 2022; Caine *et al.* 2023). We noticed that stomata PW was particularly sensitive to stress conditions compared to PL, SW, and SL and it exhibited significant variation among the genotypes. This highlights its potential as a critical trait to consider when studying abiotic stress responses of crops. In the current study, observing the behaviour of the newly formed stomata during the stress and the same stomata during the stress recovery phase was challenging due to the short timing of experimental treatments (7 days for stress and 5 days for recovery) and the damage inflicted on the leaf portions used for impressions. Furthermore, due to the severity of stress under the combined drought and heat treatment, two genotypes failed to develop fully expanded leaves within 7 days. Consequently, they could not be compared with the other genotypes for their stomatal behaviour. These potential methodological limitations should be considered in upcoming similar studies via longer stress treatment sufficient to develop new leaves. Nonetheless, it could be intriguing to investigate and compare the recovery patterns of stomata anatomy in future studies.

In the stress-tolerant Akabare chili landraces, higher rates of photosynthesis, stomatal conductance and larger biomass accumulation were kept even during stress, while sensitive genotypes showed lower rates of photosynthesis and stomatal conductance. Stress conditions, particularly the combination of drought and heat stresses significantly reduced CO_2_ gain and WUE, leading to decreased aerial and root biomass. It is in line with previous findings where combined stress was found to be the most critical factor in chili (Zamljen *et al.* 2020), tomato (Nankishore and Farrell 2016; Zhou *et al.* 2017b) and wheat (Abdelhakim *et al.* 2021). Akabare chili landraces under drought stress had comparatively lower values of responses of *F*_*v*_/*F*_*m*_, *g*_*s*_, *E*, *P*_N_, C*i*, TVDW and PASA compared to those under heat stress, indicating a higher level of severity of soil dryness over thermal stress, as observed previously in chili (Kopta *et al.* 2020) and tomato (Zhou *et al.* 2018). All genotypes in this study showed a considerable decrease in physiological responses when exposed to combined stress conditions. It can be inferred that the effects of combined stress dominate over individual stressors in controlled conditions (Chaves *et al.* 2009; Zhou *et al.* 2017*b*), and when it comes to an individual, the drought stress dominated heat stress in Akabare chili landraces too.

Drought stress is a more prevalent occurrence than heat stress in the Akabare chili cultivation areas located mostly in mid-hills regions of Nepal with altitudes ranging from 900 to 2000 m. According to a field study conducted in 2021, 33.5 % of 120 Akabare chili growers across seven locations were contending with drought stress on their chili farms, and 13.8 % for heat stress in the same year (Poudyal *et al.* 2023*c*). This study builds upon our previous research (Poudyal *et al.* 2023*c*), which marked the beginning of a scientific exploration into Akabare chili landraces under drought and heat stress. Nevertheless, to delve deeper into the underlying physiological mechanisms, numerous additional studies are required. The current study used plants at their early vegetative stage and focused on a few physiological responses of agronomic importance, however, it would be difficult to predict the plant performance in similar responses under stress conditions during the flowering and fruiting stage. Fewer studies in the past have reported mixed findings on whether tolerance to high temperature and water limitation in one developmental stage may related to tolerance at other stages (Zhou *et al.* 2017*a*; Poudyal *et al.* 2019). Further, field-based studies are crucial to understanding the impact of the changing climate on Akabare chili production. Future studies should encompass more cultivation areas, given that open field conditions make chili cultivation susceptible to multiple stresses. Our study focused on eight Akabare chili landraces out of a collection of 28 from specific geographical regions. Achieving phenological uniformity among Akabare chili landraces posed a challenge in this research. To address issues in Akabare chili landraces breeding and accelerate the integration of stress-tolerant landraces into agriculture, future research should prioritize screening numerous genotypes from diverse agroecological zones for multiple stress tolerance across various growth stages, including pre-flowering and fruiting. Furthermore, in addition to phenomics, it is advisable to incorporate ‘omics’ approaches, including genomics, proteomics and metabolomics, in future studies to gain a more profound understanding of the complexities of Akabare chili landraces when cultivated under adverse conditions.

## Conclusions

The importance of understanding the physiological responses and mechanisms to abiotic stress is emphasized by studying the sensitivity of Akabare chili landraces to drought, heat and combined stress. The study compared physiological shifts in Akabare chili landraces under four treatment conditions and the recovery phase. Both drought stress and heat stress had severe to moderately severe impacts, respectively, on the physiological processes of plants at the early vegetative stage, but their combination had an extremely severe effect. The present study revealed that Akabare chili landraces showed similar response patterns in the studied parameters as of common chili and other crops, however, landraces showed genotype-specific physiological mechanisms to cope with individual and combined stresses of drought and heat. The efficient photochemical mechanism of photosystem II photochemistry, higher transpiration rate, net photosynthetic rate, stomatal conductance, cooler leaf, wider stomata pores and WUE of tolerant landraces might have contributed to continuous growth and development during the stress condition and to accelerated recoveries from physiological damages caused by stresses. Based on the findings, landraces C44, C45C and DKT77 are found to be tolerant genotypes to individual and combined stressors and identified as potential candidates for further studies. The study revealed the genotypic variation among Akabare chili landraces and provided a theoretical basis for improving the tolerance of chili landraces to drought and heat stress. Further studies could elucidate the biochemical and molecular mechanisms behind crop tolerance to abiotic stress to achieve tangible progress in the Akabare chili breeding programme.

## Supporting Information

The following additional information is available in the online version of this article–

"Akabare chili phenotyping: Supplementary tables and figures.doc"

"Akabare chili phenotyping: Supplementary data set for the study.xls"

plad083_suppl_Supplementary_Material

plad083_suppl_Supplementary_Data

## Data Availability

Supplementary information for this article, along with the underlying study data, is available in the online version of this article. The corresponding author will address reasonable requests.
