# Supplementary material for: Evaluating the physiological responses and identifying stress tolerance of Akabare chili landraces to individual and combined drought and heat stresses
Source: AoB Plants. 2023 Nov 25;15(6):plad083. doi: 10.1093/aobpla/plad083 (PMC10721449; doi:10.1093/aobpla/plad083)
Supplement: plad083_suppl_Supplementary_Material [file plad083_suppl_supplementary_material.docx]

# Supplementary Materials

Physiological responses of Akabare chili landrace (*Capsicum annuum* L.) seedlings to drought and heat stress

## **Development and pre-evaluation of plant materials**

The supplementary Table 1 includes a list of selected landraces from two pre-evaluation trials conducted in 2019 in Thankot, Kathmandu (27.688235°N, 85.221221°E), and 2020 in Thankot, Kathmandu and Sanghar, Salyan (28.336786°N. 82.231586°E). Chili variety 'Jwala' was not included in the pre-evaluations but was used as a standard check in stress phenotyping experiments. Of the total 28 landraces, 8 were obtained from the National Agriculture Resources Center (Gene bank), Lalitpur, Nepal (27.647038°N, 85.324064°E), and 20 were collected from various districts of Nepal as shown in the Table 1. The SEAN Seed Service Center Limited (Sean Seed), located in Thankot, Kathmandu, Nepal, supplied the Jwala variety. Due to their similar phenology, a total of sixteen landraces from the 2019 trials were selected for further homogeneity purification. Of these, eight landraces, which exhibited consistent morphology, growth habits, and higher fruit yield in 2020, were retained at the Thankot, Kathmandu location. The seeds from these eight landraces were combined into a bulk for use in subsequent phenotyping experiments.

Supporting Information⎯**Table S1**: Details of plant materials used in the study. Original name at source, place of collection, codes used during the experiment, GPS coordinates of collection center are listed.

| **S. N.** | **Local/given name** | **Code** | **Source** | **GPS Coordinates** | **2019 trial results**  **(16 landraces selected)** | **2020 trial results**  **(8 landraces selected)** |
| --- | --- | --- | --- | --- | --- | --- |
| 1 | Golo Akabare | BJ77 | Fikkal, Ilam | 26.895297°N, 88.068550°E | Selected | Selected |
| 2 | Dalle Akabare | DA77 | Aathghare, Ilam | 26.920271°N, 88.064594°E | Selected |  |
| 3 | Gaithune Akabare | IL76 | Aathghare, Ilam | 26.920271°N, 88.064594°E | Selected |  |
| 4 | Thulo Akabare | AK77 | Fikkal, Ilam | 26.895297°N, 88.068550°E |  |  |
| 5 | Bayere Akabare | BA77 | Fikkal, Ilam | 26.895297°N, 88.068550°E |  |  |
| 6 | CO11044 | C44 | Gene bank | 27.647038°N, 85.324064°E | Selected | Selected |
| 7 | CO11045 | C45C* | Gene bank | 27.647038°N, 85.324064°E | Selected | Selected |
| 8 | CO11062 | C62 | Gene bank | 27.647038°N, 85.324064°E | Selected | Selected |
| 9 | CO11064 | C64B* | Gene bank | 27.647038°N, 85.324064°E | Selected | Selected |
| 10 | CO11064 | C64C* | Gene bank | 27.647038°N, 85.324064°E | Selected | Selected |
| 11 | CO11046 | C46 | Gene bank | 27.647038°N, 85.324064°E |  |  |
| 12 | CO11047 | C47 | Gene bank | 27.647038°N, 85.324064°E |  |  |
| 13 | CO11054 | C54 | Gene bank | 27.647038°N, 85.324064°E |  |  |
| 14 | Dhankute Akabare | DKT77 | Hile, Dhankuta | 27.031459°N, 87.312967°E | Selected | Selected |
| 15 | Ghopte Akabare | DKS77 | Sidhuwa, Dhankuta | 27.078826°N, 87.389324°E | Selected |  |
| 16 | Dalle Akabare | DKH77 | Hile, Dhankuta | 27.031459°N, 87.312967°E |  |  |
| 17 | Jwala | Jwala** | Sean Seed | 27.688235°N, 85.221221°E | Not included | Not included |
| 18 | Ghopte Akabare | PPR77 | Rankebazzar, Panchthar | 27.0295142°N, 87.805548°E | Selected | Selected |
| 19 | Chuche Akabare | PRR77 | Rankebazzar, Panchthar | 27.0295142°N, 87.805548°E |  |  |
| 20 | Thepche Akabare | PRP77 | Rankebazzar, Panchthar | 27.0295142°N, 87.805548°E | Selected |  |
| 21 | Gaithune Akabare | GT77 | Sukrabare, Tehrathum | 27.056998°N, 87.450288°E |  |  |
| 22 | Bagale Akabare | DT77 | Sukrabare, Tehrathum | 27.056998°N, 87.450288°E | Selected |  |
| 23 | Madane Akabare | LT77 | Sukrabare, Tehrathum | 27.056998°N, 87.450288°E |  |  |
| 24 | Punte Akabare | PA77 | Khatigaunda, Dailekh | 28.793368°N, 81.604840°E | Selected |  |
| 25 | Dalle Akabare | PD77 | Khatigaunda, Dailekh | 28.793368°N, 81.604840°E |  |  |
| 26 | Dalle Akabare | DC77 | Chitlang, Makawanpur | 27.632094°N, 85.164200°E | Selected |  |
| 27 | Bayere Akabare | BB77 | Nagarkot, Bhaktapur | 27.710803°N, 85.494384°E |  |  |
| 28 | Ghopte Akabare | BN77 | Nagarkot, Bhaktapur | 27.710803°N, 85.494384°E | Selected |  |

* The variants segregated from CO11064 were classified as C45C, C64B and C64C depending on the shape of their fruits.

** Chili variety 'Jwala' was not included in the pre-evaluation trials.

## **Biomass accumulation under the stress conditions and recovery phase**

The values of total vegetative fresh weight (TVFW), shoot dry weight (ShDW), leaf dry weight (LDW), and leaf mass per leaf area (LMA) were significantly affected by stress conditions, and there were noticeable differences in their values among the different genotypes under specific treatment conditions (Supplementary Figure 1). The combined stress of drought and heat (DHT) reduced TVFW by 50% followed by drought (29%) and heat stress (15%) during stress treatment (Supplementary Figure 1A). However, during the recovery, this difference was small however significant compared to the control values; 20% was for combined stress, 14% for drought, and 9% was for recovery from heat stress. Similar patterns were seen for ShDW, LDW and LMA (Supplementary Figure 1B, C, D). The DHT significantly reduced ShDW, LDW, and LMA of genotypes in the range of 27% to 63% compared to control. Drought stress (DT) was found to be more detrimental to LDW and LMA than heat stress (HT). In terms of biomass production and partitioning, landrace C44 performed the best on average across all treatments, followed by C45C and DKT77. On the other hand, landraces C64C, PPR77, and variety Jwala were among the poorest performers among the nine genotypes (Supplementary Figure 1A, B, C and D).

**
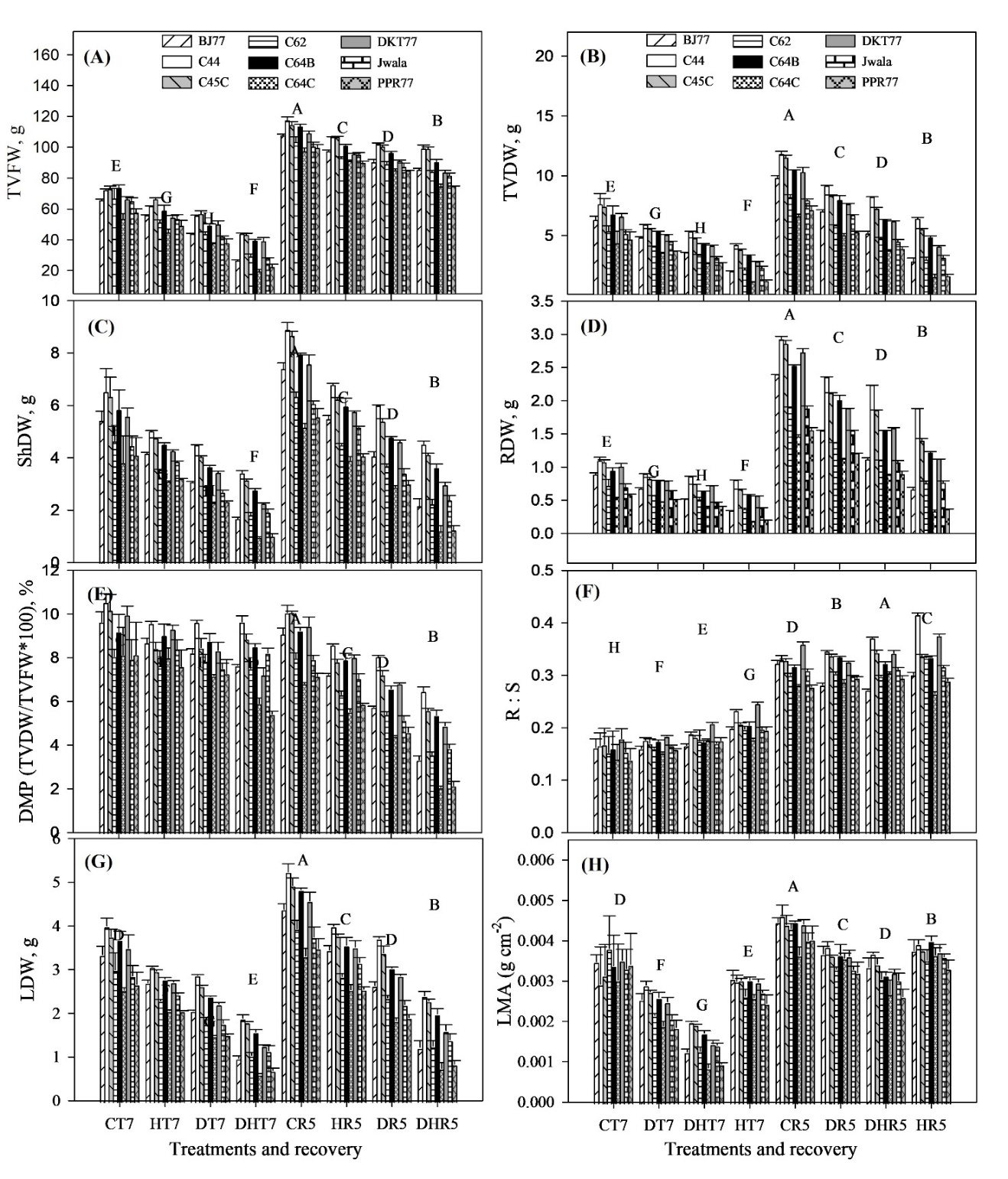
**

Supporting Information⎯Figure S1: Fresh and dry biomass production and partitioning. The biomass of evaluated eight Akabare chili landrace and Jwala measured at the end of treatment, treatment day 7 (T7), and the recovery phase, recovery day 5 (R5). (A) Total vegetative fresh weight (TVFW), g; (B) Shoot dry weight (ShDW), g; (C) Leaf dry weight (ShDW), g; and (D) Leaf mass per area (LMA), g cm^-2^. CT denotes control, DT drought stress, HT heat stress and DHT denotes combined drought, and heat stress treatment, and CR, DR, HR and DHR denotes recovery of respective treatment. Data are mean values ± *SD* (n = 3). Different letters stand for signiﬁcant differences (*p* < 0.05).

# Stress increases heat injury index and wilting scores in susceptible genotypes.

Under stress conditions, Jwala and C64C had significantly higher wilting score (WS) values, while the lowest WS value was observed in C44 (Supplementary Table 3). Additionally, compared to the other seven genotypes, C44 and C45C had significantly lower heat injury index (HII) values (Supplementary Table 3). The variation in WS and HII recovery among the genotypes was clear during the recovery phase.

Supporting Information⎯Table S2: Plant scores recorded under drought and heat stress. The assessment of stress injury was conducted using the heat injury index (HII) and wilting score (WS), under different stress conditions including drought (DT), heat (HT), and a combination of both stresses (DHT), as well as during the recovery phase. The mean values of HII and WS were calculated for twelve independent observations under stress conditions, and for six independent observations during the recovery phase. Statistically significant differences between mean values ± *SD* within the column (p < 0.05) are denoted by different small letters.

| **Genotype** | **Stress conditions** | | **Recovery phase** | |
| --- | --- | --- | --- | --- |
|  | ***WS*** | ***HII*** | ***WS*** | ***HII*** |
| BJ77 | 2.83 ± 1.34 a | 2.58 ± 1.73 a | 2.25 ± 0.97 ab | 2 ± 1.13 ab |
| C44 | 1.92 ± 0.99 d | 1.98 ± 0.99 c | 1.58 ± 0.67 c | 1.25 ± 0.45 e |
| C45C | 2.33 ± 1.07 c | 2 ± 1.21 bc | 1.75 ± 0.75 c | 1.33 ± 0.49 de |
| C62 | 2.75 ± 1.29 ab | 2.67 ± 1.78 a | 2.25 ± 0.87 ab | 1.92 ± 0.99 ab |
| C64B | 2.42 ± 1.08 c | 2.1 ± 1.24 b | 2.08 ± 0.79 b | 1.55 ± 0.82 cd |
| C64C | 2.92 ± 1.38 a | 2.67 ± 1.83 a | 2.42 ± 0.99 a | 2.08 ± 1.16 a |
| DKT77 | 2.5 ± 1.09 bc | 2.25 ± 1.48 b | 2.08 ± 0.79 b | 1.75 ± 0.97 bc |
| Jwala | 2.92 ± 1.51 a | 2.58 ± 1.73 a | 2.32 ± 0.98 ab | 2.08 ± 1.16 a |
| PPR77 | 3 ± 1.48 a | 2.67 ± 1.78 a | 2.33 ± 0.98 ab | 2.08 ± 1.16 a |

# Stress condition increases flavonol content index and decreases leaf water content and chlorophyll content index.

The combined stress (DHT) significantly decreased the leaf relative water content (RWC) of the leaf (Supplementary Figure 2A). Compared to the control, a significant reduction of RWC by 18%, 12%, and 24% under drought, heat, and DHT, respectively, was seen. A similar trend of gain in RWC was detected during the recovery phase.

Chlorophyll content index (CCI) was significantly reduced by stress treatments (Supplementary Figure 2B). The CCI was reduced by 14% under DHT and by 8% under HT, compared to the control condition. However, the values for control and drought stress did not show significant differences. A similar trend was observed during recovery, where the CCI values for control and drought were significantly higher than heat and combined stress during the recovery phase. The highest CCI was recorded for landrace C44 and the lowest for landrace PPR77, averaged for the treatments and recovery phase.

Compared to other treatments, the flavonol content index (FCI) increased significantly under the DHT (Supplementary Figure 2C). The FI was approximately 34% higher in the DHT, 24% in HT and 22% higher in DT conditions compared to the control. However, such differences in FCI values significantly decreased as seedlings recovered from stress. Seedlings recovering DT showed 5% lower values than control, so as 4% in HT and 28% in DHT. Genotype C64B exhibited the highest FI value, while the lowest was recorded for landrace PPR77, with an average calculated throughout the treatment and recovery phase.


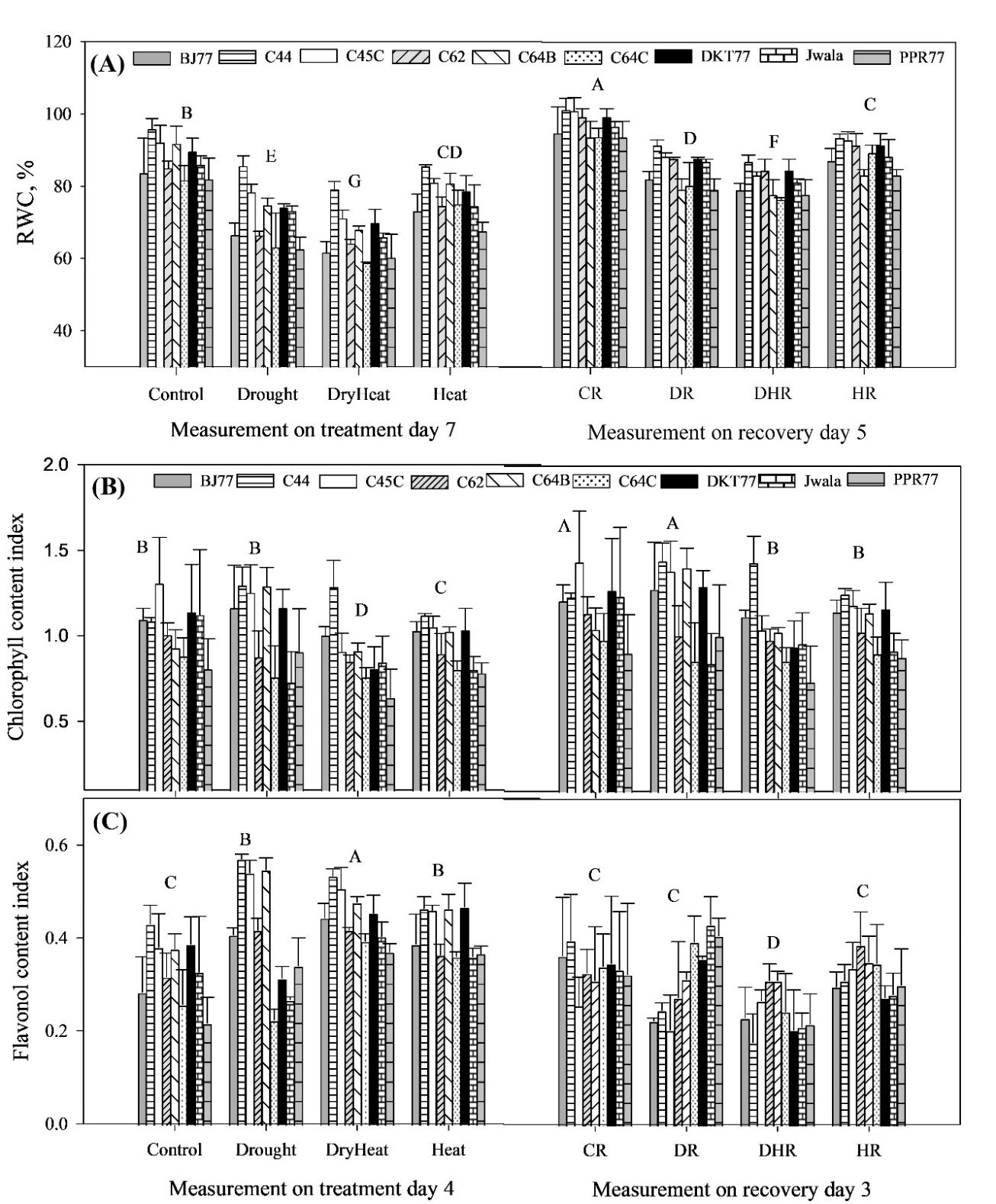


Supporting Information⎯Figure S2: Leaf water status and pigment content indices. (A) Leaf relative water content (RWC), (B) Leaf chlorophyll content index, and (C) Leaf flavonol content index of landraces and Jwala variety during treatments and recovery. RWC was assessed on day 7 (T7) and day 5 (R5) of the recovery phase. In the X-axis of (a) and (c), ControlR stands for the normal condition continued during recovery, DroughtR denotes recovering seedlings from drought stress, and so on under control condition. Data are mean ± *SD* (n = 8) and different letters show significant differences (*p* < 0.05).

# Variation in stomata size, pore size and stomata density

Stomata width (SW) was reduced by 3%, 22%, and 44% under heat, drought, and combined with both stresses compared to control (Supplementary Figure 3A). There were significant differences in SW values between genotypes even during the recovery phase. The moderate reduction was noticeable under the recovery phase also where HT, DT, and DHT had lower SW by 12%, 9%, and 24%, respectively. On average, in all treatments, the widest stomata were observed for landrace C44 and the narrowest stomata for Jwala. Under drought, heat, and DHT, the length of the stomata (SL) was reduced by 3%, 22%, and 42%, respectively, compared to the control, however, no significant difference was observed between the SL values of control and heat stress (Supplementary Figure 3B). The difference in SL was also observed during the recovery phase, with drought stress reducing it by 15% and combined stress by 32% compared to the control, however, no significant SL reduction was observed for seedlings recovering from heat stress. Among the nine, C44 and BJ77 showed the longest stomata on average, while C64B had the shortest.

The stomata pore length (PL) was found to be reduced by 13% and 39% under DT and DHT compared to the control values, but it was increased by 7% under heat stress (Supplementary Figure 3C). Across all treatments, the landraces C44 and BJ77 had the longest stomata pores, while C64B had the shortest pores, followed by PPR77 among the nine genotypes. Under heat, drought, and combined stresses, the stomata pore width (PW) was significantly reduced by 13%, 32%, and 61%, respectively, compared to the control (Supplementary Figure 3D). This difference was also observed in the recovery phase, where HR, DR, and DHR resulted in a significantly lower PW by 14%, 29%, and 59%, respectively. Among all treatments, C44 and BJ77 showed the widest pores, while C64B and PPR77 showed the narrowest pores of the stomata among the nine chili genotypes.


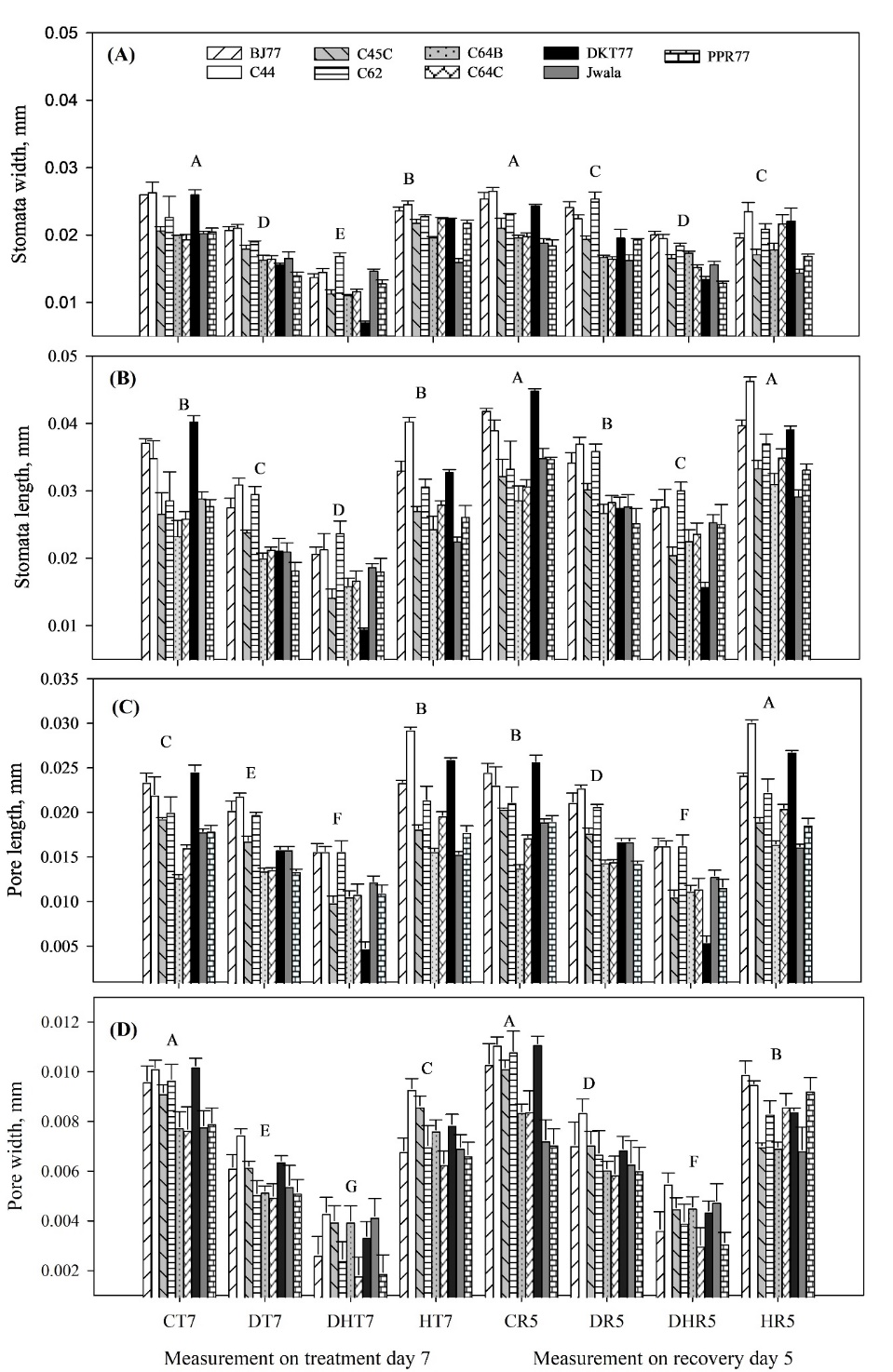


Supporting Information⎯Figure S3: Stomatal anatomy of eight Akabare chili landrace and Jwala variety grown under four treatment conditions and stress recovery phase. Imprints were independently obtained for the stomatal anatomy on stress treatment day 7 (T7) of the respective treatment and recovery phase day 5 (R5). In the figure, (A) Stomata width, mm; (B) Stomata length, mm; (C) Stomata pore length, mm; and (D) Stomata pore width, mm. CT denotes control, DT drought stress, HT heat stress and DHT denotes combined drought, and heat stress treatment, and CR, DR, HR and DHR denotes recovery of respective treatment. Data are mean± *SD* (n = 10) and the different letters above the bars show significant differences (*p* < 0.05).


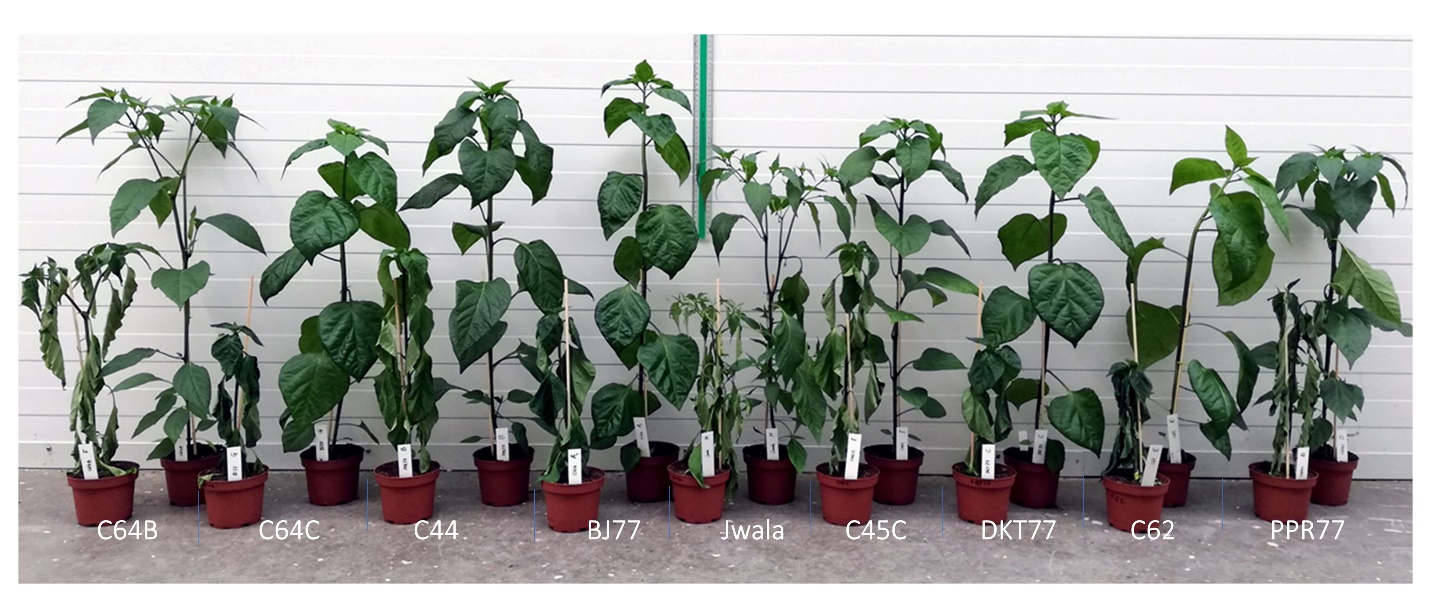


Supporting Information⎯Figure S4: Chili seedlings under the different treatment condition. Seedlings under control (taller, right side) and drought stress treatment (shorter, left side) condition are shown with label. Chili seedlings under the control (taller, right side) and drought stress treatment (shorter, left side). The figure displays two seedlings per genotype, totaling nine genotypes as labeled in the figure. The photo was taken prior to destructive measurement after stress treatment.


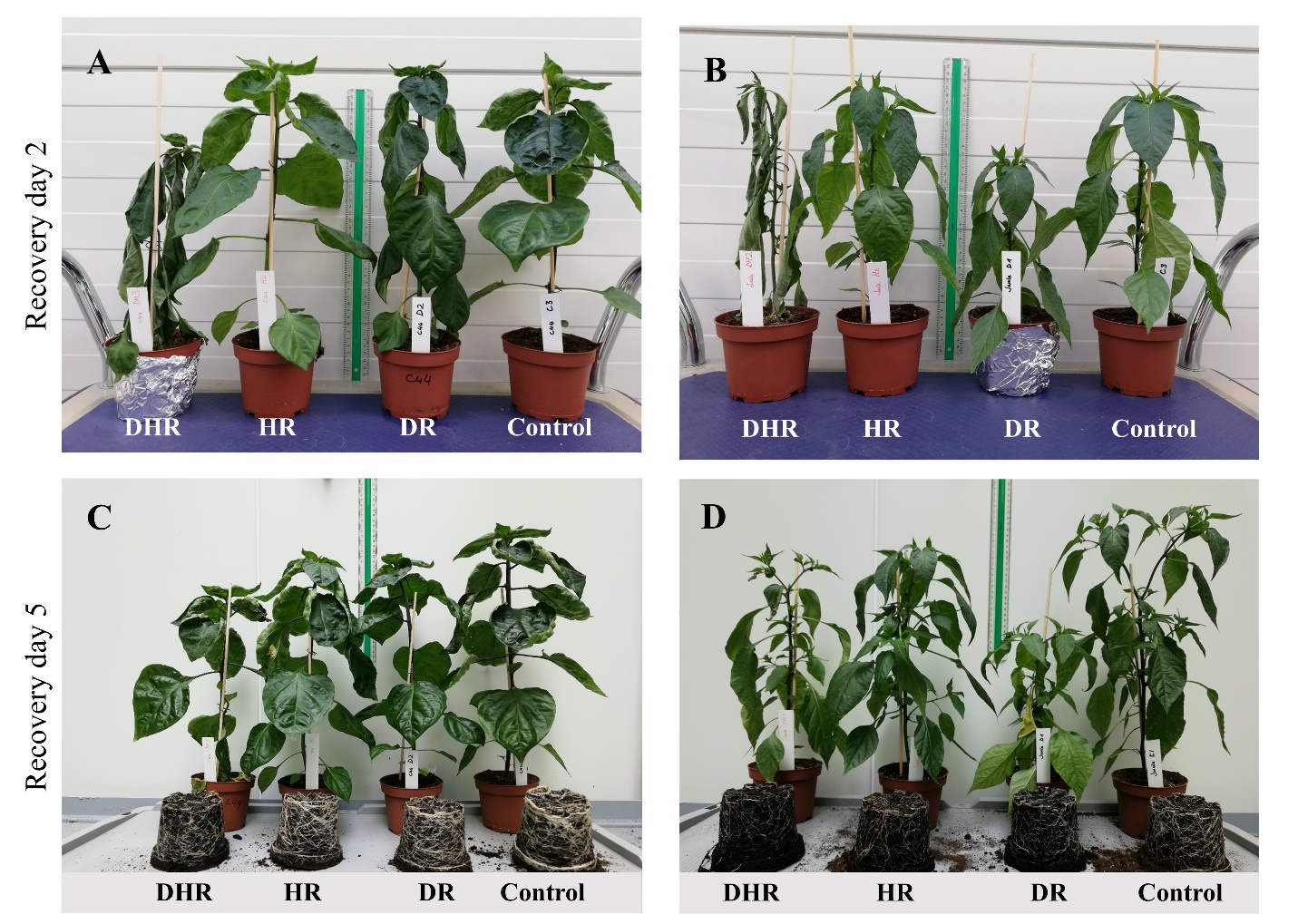


Supporting Information⎯Figure S5: Chili plants under recovery phase. Top row: (A) Plant morphology of Akabare chili landrace (C44), and (B) chili variety ‘Jwala’, recovering from stress conditions (DHTR: recovery from combination of drought and heat stress, HTR: recovery from heat stress, DTR: recovery from drought stress, and control condition) during the recovery phase day 2; and bottom row: (C) plant morphology of C44 and (D) Jwala, recovering from stress conditions under recovery phase (recovery day 5).


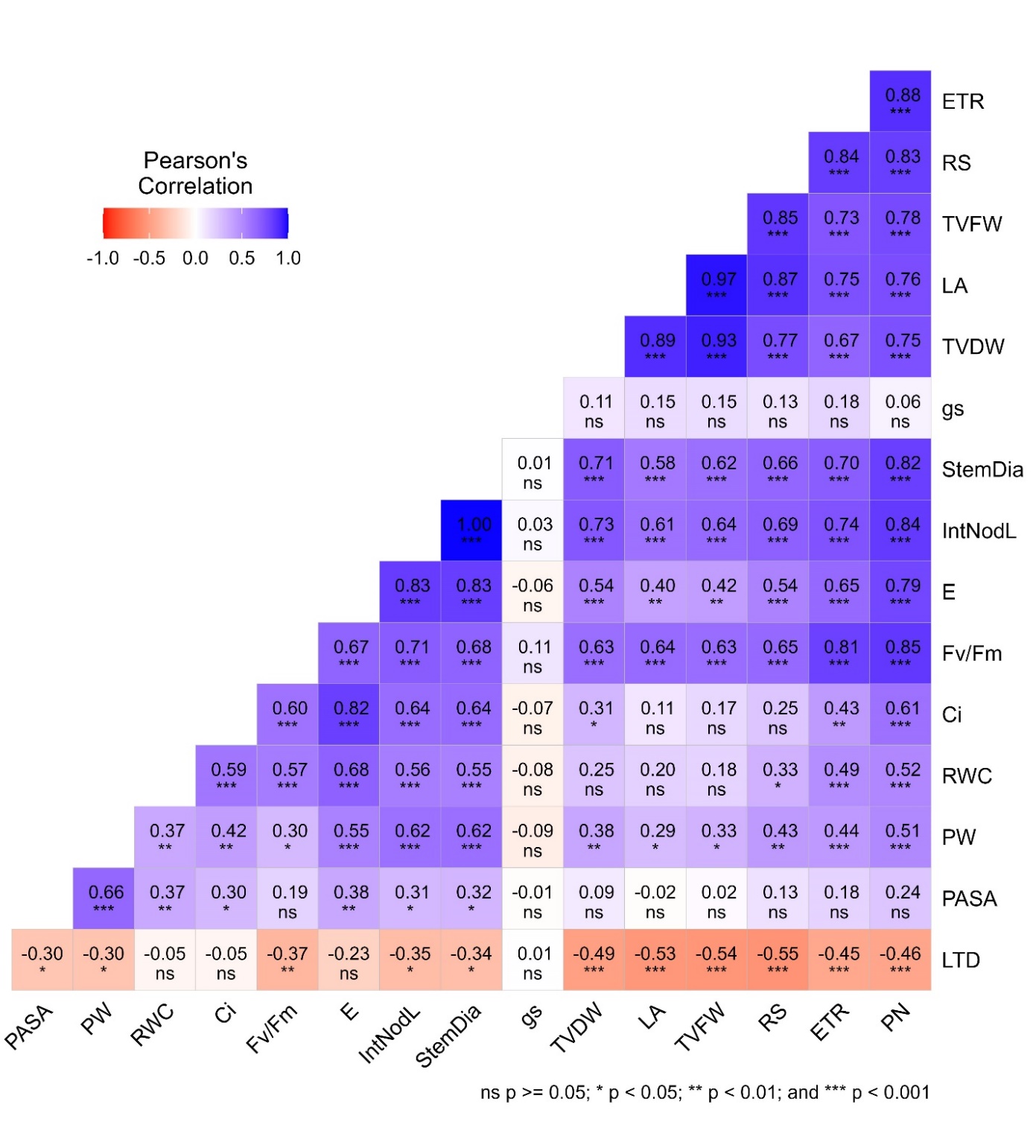


Supporting Information⎯Figure S6: A correlation matrix plot illustrating the interrelationships between 16 morphological and physiological traits observed under control condition. PASA, ratio of stomata pore area to stomata area; PW, stomata pore width; RWC, leaf relative water content; Ci, intercellular CO_2_ concentration; F_v_/F_m_, chlorophyll fluorescence; E, transpiration rate; IntNodL, internode length; StemDia, stem diameter; g_s_, stomata conductance; TVDW, total vegetative dry weight; LA, leaf area; TVFW, total vegetative fresh weight; RS, root to shoot dry weight ratio; ETR, electron transfer rate; P_N_, net photosynthesis; and LTD, leaf temperature depression. ns, nonsignificant; *, p ≥ 0.05; **, p ≥ 0.01; ***, p ≥ 0.001 level.


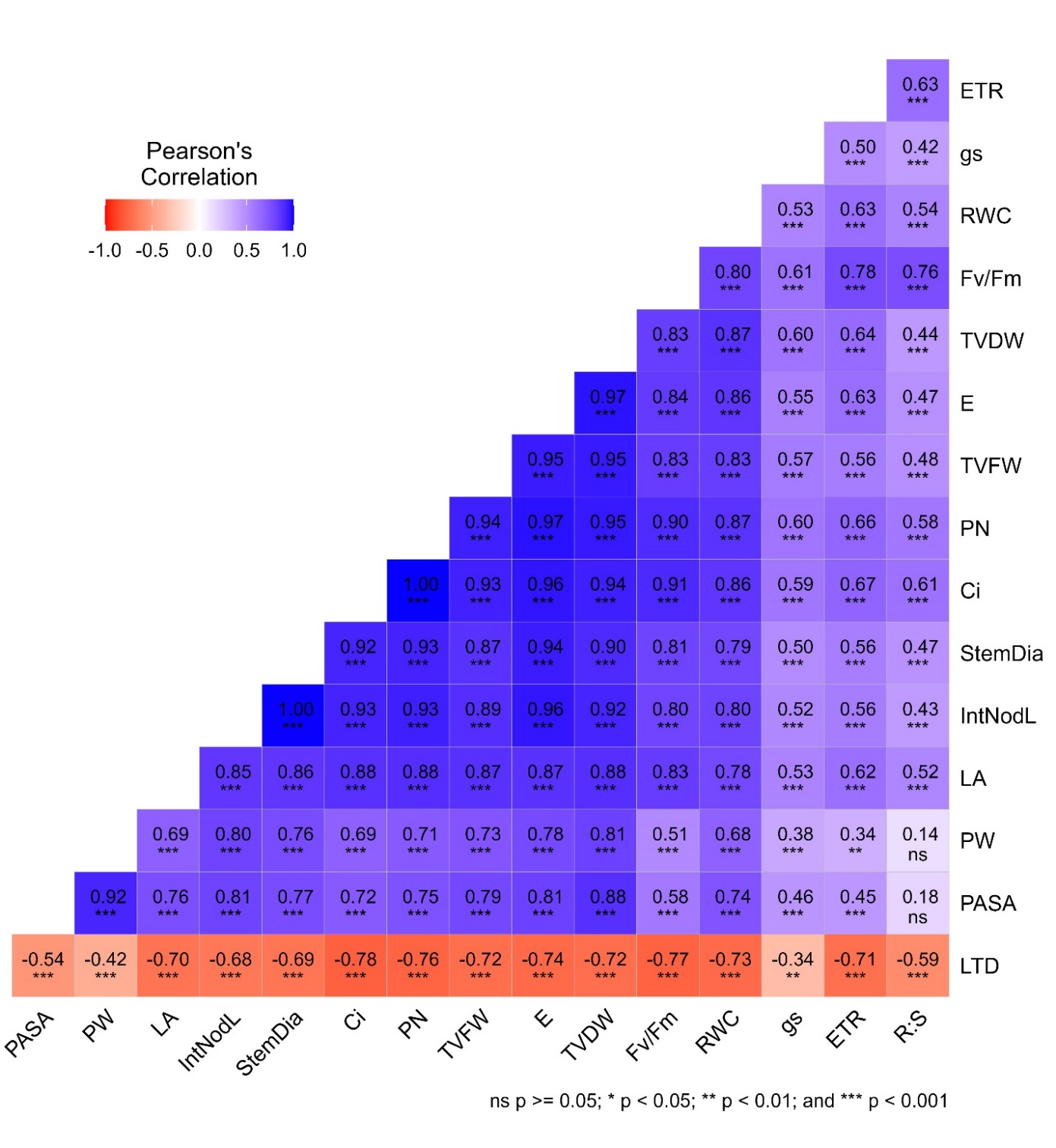


Supporting Information⎯Figure S7: A correlation matrix plot illustrating the interrelationships between 16 morphological and physiological traits observed under recovery condition. In figure: PASA, ratio of stomata pore area to stomata area; PW, stomata pore width; LA, leaf area; IntNodL, internode length; StemDia, stem diameter; Ci, intercellular CO_2_ concentration; ; P_N_, net photosynthetic rate; TVFW, total vegetative fresh weight; E, transpiration rate; TVDW, total vegetative dry weight; F_v_/F_m_, chlorophyll fluorescence; RWC, leaf relative water content; g_s_, stomata conductance; ETR, electron transfer rate; R:S, root to shoot dry weight ratio; and LTD, leaf temperature depression. ns, nonsignificant; *, p ≥ 0.05; **, p ≥ 0.01; ***, p ≥ 0.001 level.


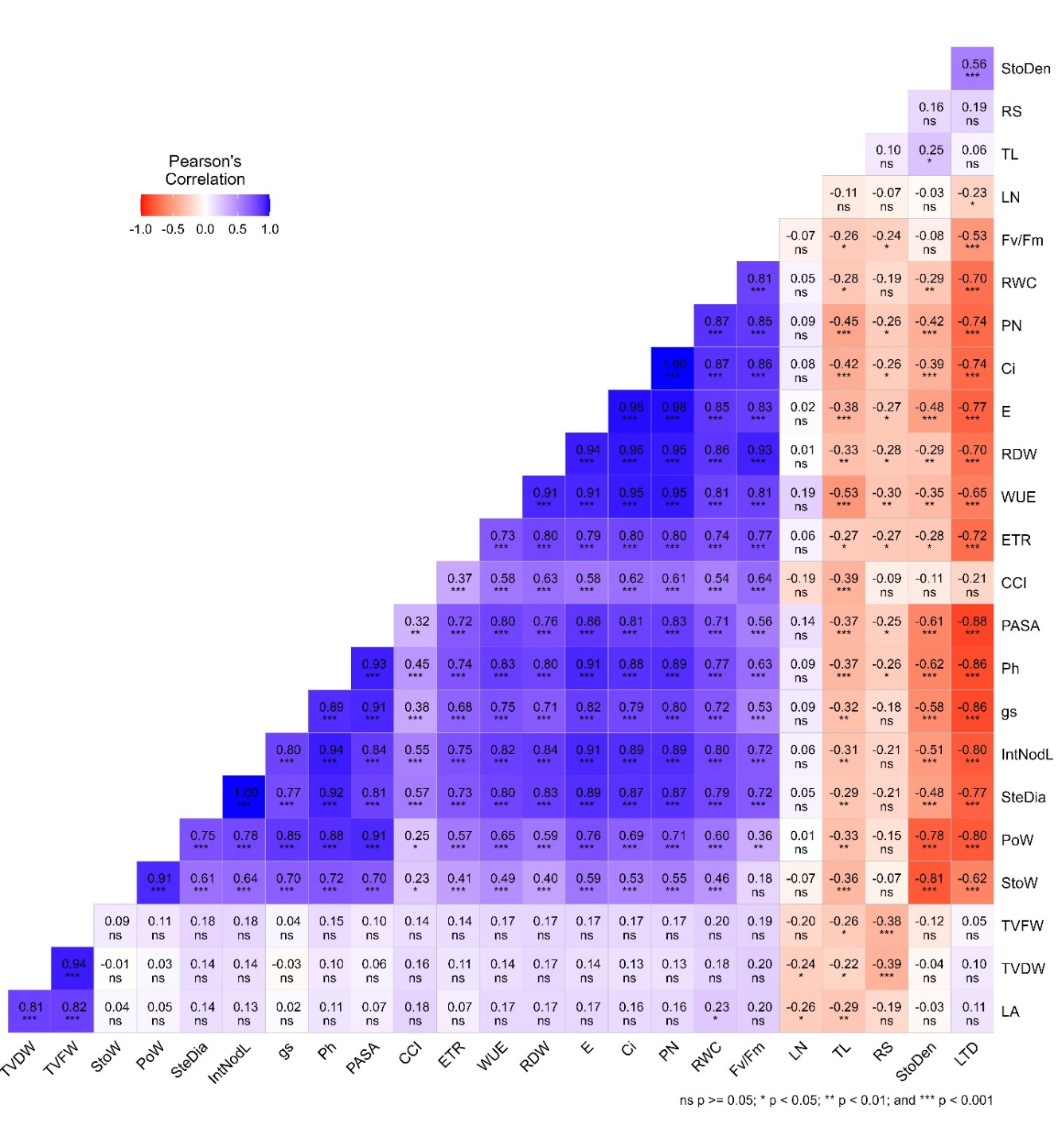


Supporting Information⎯Figure S8: A correlation matrix plot illustrating the interrelationships between 24 morphological and physiological traits observed under stress (drought, heat and combined of both) condition. In figure: TVDW, total vegetative dry weight; TVFW, total vegetative fresh weight; StoW, stomata width; PoW, stomata pore width; SteDia, stem diameter; IntNodL, internode length; g_s_, stomata conductance; Ph, plant height; PASA, ratio of stomata pore area to stomata area; CCI, chlorophyll content index; ETR, electron transfer rate; WUE, water use efficiency; RDW, root dry weight; E, transpiration rate; Ci, intercellular CO_2_ concentration; P_N_, net photosynthesis; RWC, leaf relative water content; F_v_/F_m_, chlorophyll fluorescence; LN, leaf number; T_L_, leaf temperature; RS, root to shoot dry weight ratio; StoDen, stomata density; LTD, leaf temperature depression; and LA, leaf area. ns, nonsignificant; *, p ≥ 0.05; **, p ≥ 0.01; ***, p ≥ 0.001 level.

Supporting Information⎯Table S3: Selected significant variables contributing to total biomass production from stepwise multiple regression analysis. Total vegetative dry weight (TVDW) was considered a dependent variable. Regression analysis was conducted using sixteen morphological and physiological traits. Coefficients, standard error, t- stat and *p* values are shown. Response variable is TVDW, total vegetative dry weight, and contributing variables with no collinearity are: LTD, leaf temperature depression; T_L_, leaf temperature; SD, stomata density (under control treatment); LA, leaf area (under stress treatment); and LN, leaf number; WS, wilting score (under recovery phase). SEE, standard error of estimate; VIF, variance inflation factor.

| Model | Condition | Regression statistics | | | | |  |
| --- | --- | --- | --- | --- | --- | --- | --- |
|  |  | Parameters | Coefficient | Standard Error | t Stat | *p*-value | VIF |
| *TVDW = -0.833 - (2.154 × LTD) - (0.174 × TL) - (0.00474 × SD)*  N = 54, R = 0.592, R^2^ = 0.350, Adj R^2^ = 0.311, SEE = 2.267 | Control | Constant | -0.833 | 7.058 | -0.118 | 0.906 | - |
|  |  | LTD | -2.154 | 0.568 | -3.794 | <0.001 | 1.097 |
|  |  | TL | -0.174 | 0.0841 | -2.072 | 0.043 | 1.084 |
|  |  | SD | -0.00474 | 0.00230 | -2.063 | 0.044 | 1.016 |
| TVDW = -7.952 + (0.0133 *×* LA)  N = 81, R = 0.814, R^2^ = 0.663, Adj R^2^ = 0.658, SEE = 0.824 | Stress | Constant | -7.952 | 0.958 | -8.301 | <0.001 | - |
|  |  | LA | 0.0133 | 0.00107 | 12.460 | <0.001 | 1.000 |
| TVDW = 8.332 - (2.080 *×* WS) + (0.0308 *×* LN)  N = 81, R = 0.760, R^2^ = 0.578, Adj R^2^ = 0.567, SEE = 1.324 | Recovery | Constant | 8.332 | 1.098 | 7.586 | <0.001 | - |
|  |  | WS | -2.080 | 0.216 | -9.646 | <0.001 | 1.014 |
|  |  | LN | 0.0308 | 0.0121 | 2.550 | 0.013 | 1.014 |
